# A new intronic quantitative PCR method led to the discovery of transformation from human ascites to murine malignancy in a mouse model

**DOI:** 10.3389/fonc.2023.1062424

**Published:** 2023-02-06

**Authors:** Jiankang Jin, Longfei Huo, Yibo Fan, Ruiping Wang, Ailing W. Scott, Melissa Pool Pizzi, Xiaodan Yao, Shan Shao, Lang Ma, Matheus S. Da Silva, Kohei Yamashita, Katsuhiro Yoshimura, Boyu Zhang, Jingjing Wu, Linghua Wang, Shumei Song, Jaffer A. Ajani

**Affiliations:** ^1^ Department of Gastrointestinal Medical Oncology, The University of Texas MD Anderson Cancer Center, Houston, TX, United States; ^2^ Department of Genomic Medicine, The University of Texas MD Anderson Cancer Center, Houston, TX, United States

**Keywords:** intronic genomic qPCR, authentication and quantification of human and murine samples, patient-derived xenograft (PDX), human-to-murine oncogenic transformation, whole exosome sequencing (WES), tumor microenvironment (TME)

## Abstract

**Purpose:**

To establish a fast and accurate detection method for interspecies contaminations in the patient-derived xenograft (PDX) models and cell lines, and to elucidate possible mechanisms if interspecies oncogenic transformation is detected.

**Methods:**

A fast and highly sensitive intronic qPCR method detecting Gapdh intronic genomic copies was developed to quantify if cells were human or murine or a mixture. By this method, we documented that murine stromal cells were abundant in the PDXs; we also authenticated our cell lines to be human or murine.

**Results:**

In one mouse model, GA0825-PDX transformed murine stromal cells into a malignant tumorigenic murine P0825 cell line. We traced the timeline of this transformation and discovered three subpopulations descended from the same GA0825-PDX model: epithelium-like human H0825, fibroblast-like murine M0825, and main passaged murine P0825 displayed differences in tumorigenic capability *in vivo*. P0825 was the most aggressive and H0825 was weakly tumorigenic. Immunofluorescence (IF) staining revealed that P0825 cells highly expressed several oncogenic and cancer stem cell markers. Whole exosome sequencing (WES) analysis revealed that TP53 mutation in the human ascites IP116-generated GA0825-PDX may have played a role in the human-to-murine oncogenic transformation.

**Conclusion:**

This intronic qPCR is able to quantify human/mouse genomic copies with high sensitivity and within a time frame of a few hours. We are the first to use intronic genomic qPCR for authentication and quantification of biosamples. Human ascites transformed murine stroma into malignancy in a PDX model.

## Graphical Abstract

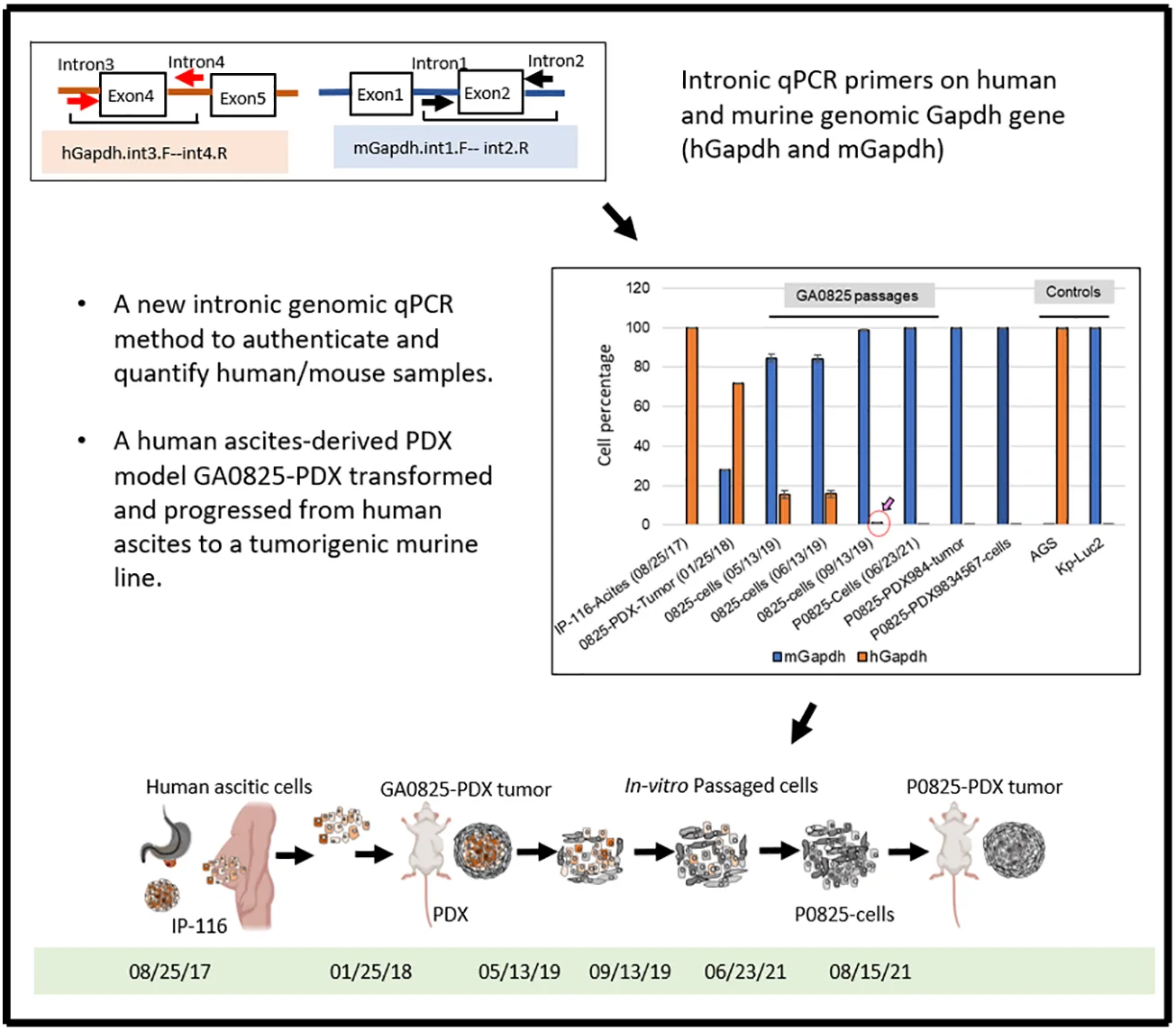

## Introduction

The patient-derived xenograft (PDX) model is a ubiquitous platform in cancer research that includes biomarker development, drug testing, and development (1). Tumor fragments or malignant ascites cells can be surgically transplanted into severe combined immune deficient (SCID), nude, or other immune-compromised mice to generate the PDX models. In some circumstances, PDX susceptibility to drugs may be closely correlated with responses in patients. Despite the limitations of the PDX models, they provide useful information in certain contexts (2). A total of 49 colorectal carcinoma-derived PDXs were used for drug testing and validated by experiments. Sensitivity of PDXs to chemotherapy and multiple targeted drugs revealed higher levels of responses corresponding to patient experiences (3). The PDX models provide complementary information to *in vitro* cell culture models (4).

In PubMed, there are few reports of interspecies host contamination in the PDX models by searching variable key words. According to our institutional Cytogenetics and Cell Authentication Core (CCAC), among the cell lines sent for cytogenetic G-banded karyotyping analyses, contamination rate of host cells in the PDX-derived cells was 39.1% (unpublished data). A human-derived prostate tumor cell line in athymic nude mice produced human, murine stromal and mixed human/murine cells, suggesting that cancer cells are capable of transforming host stromal cells (5). Later on, three murine cancer cell lines had been induced by the injection of three different human prostate cancer cell lines, which revealed selective amplification of the Y chromosome (6). They had unique marker chromosomes involving mouse chromosome 12, with a common break point. These observations further confirmed that human prostate tumors are capable of transforming host cells (7).

A PDX Authentication System (PAS) using single nucleotide polymorphisms (SNPs) to validate PDXs established murine contamination with sensitivity of 3% in individual mice from acute lymphoblastic leukemia (ALL) biopsies (8), similar to that of short tandem repeat (STR) profiling (9). Using a species-specific PCR amplicon length (ssPAL) analysis for genomic quantification of murine stroma in PDXs, it was reported that in the lung cancer PDX lines, murine stromal contamination exhibited a few to more than 95% (10). STR and SNP assays are widely used with sensitivity only at 5%–10% and 3%–5%, respectively. A barcoded deep next generation sequencing (NGS)-based method was used to authenticate human and mouse biosamples with 0.1% sensitivity and could profile 100–200 samples in a single run (11). For the NGS analyses of WES and RNAseq expression in the PDX samples currently available analysis methodologies, Xenome, Disambiguate, bamcmp and pdxBlacklist were compared to address this murine contamination issue (12). Contaminating host reads from PDXs and circulating tumor cell-derived explant (CDX)-derived WES and RNAseq data was a major confounding factor (13). To overcome contamination by the host organisms, a platform was developed for imaging cytometry (marking EpCam) integrated with micropallet array technology (14). In cultured cell lines, karyotyping and isozymes analyses revealed interspecies contaminants in a minority of cases (9%, 33 out of 360 lines) (15). Therefore, for authentication and quantification, STR, SNP, flow cytometry, and NGS techniques are used. STR and SNP are with less sensitivity, flow cytometry by using such as EpCam suffers cross-reactivity of antibodies, and NGS is more sensitive but with high cost burden.

Currently there are mainly two methods to authenticate cell lines: G-banded karyotyping and STR analysis (or the so-called DNA fingerprinting). G-banded karyotyping uses Giemsa stain on chromosomal banding of heterochromatin and euchromatin to identify chromosomes, usually it counts 20 metaphase cells. This karyotyping is a widely used genetic assay that detects chromosomal abnormalities (16), which can authenticate either human or murine cells but may be problematic if mixed human/mouse cells are analyzed. STR analysis is based on the variable number tandem repeats (VNTR) from genomic minisatellite DNAs that hybridize to many loci throughout the genome to produce DNA fingerprinting (17). The STR DNA patterns are searched against the existing databases. Normally only the human STR service is available, not for mice; moreover, STR authentication is to ask if derived cells are truly descended from the original cell lines or not. Both G-banded karyotyping and STR analysis do not work if the samples are mixed populations, particularly if they are tumors not cells. Both services are provided by professionals in core facilities or companies and take weeks if not months to finish.

The tumor microenvironment (TME) has complex cross-talks between tumor cells within PDXs. The tumor stroma is a critical component of the TME, where it has crucial roles in tumor initiation, progression, and metastases. Tumors comprise of cancer cells as well as a stroma of cellular and noncellular components, such as CAF (cancer-associated fibroblast) cells, MSC (mesenchymal stem cells), ECM (extracellular matrix), and others. After three to five passages, when the PDX models can be used for drug screening, the human tumor-associated stroma is almost completely replaced by murine-derived ECM and fibroblasts. This new murine stroma is likely to cause drastic changes in immunologic regulation of the tumors as well as in its physical properties (2). Cancer cells also release bioactive DNAs into the cytosol *via* extracellular vesicles (EVs) and extracellular particles (EPs) (18). Also, exosomes shuffle from donor cells to target cells *via* endocytosis or multivesicular bodies (MVB) which include proteins, nucleic acids, and lipids (19).

In this study, we report that a simple quantitative PCR (qPCR) by measuring intronic genomic Gapdh copies of human Gapdh (hGapdh) and murine Gapdh (mGapdh) that can quickly quantify if a tumor or a cell line is of human or murine origin, or a mixture with high sensitivity and within a time frame of a few hours. This method does not quantify mRNA-expressed cDNA copies, but only genomic copies. To our best knowledge, this is the first report to use intronic genomic qPCR to determine and quantify if a PDX tumor or a human cell line has been cross-contaminated by murine tissue or cells, or *vice versa*. Moreover, by this intronic genomic qPCR, we have identified that a PDX model GA0825-PDX generated by a gastric cancer patient’s ascites underwent transformation unexpectedly from a purely human ascites (IP-116) to a purely aggressively tumorigenic murine line P0825 over passages. WES analysis of this transformation detected only one somatic TP53 mutation in the whole genome of this GA0825-PDX, which may have played a role in a plethora of other mutations, including oncogenes, tumor suppressors, and growth factors whose genomic instability and alterations may have contributed to this human-to-mouse oncogenic transformation.

## Materials and methods

### Cancer cell lines, patient ascites, and their murine PDX models

Gastric cancer cell lines AGS, GT5, KatoIII, MKN45, N87, YCC1, YCC2, Snu1, and Snu16 were described earlier (20). Kp-luc2 murine GAC cell line was from Dr. Jo Ishizawa in the Department of Leukemia at MD Anderson Cancer Center and was previously reported (21). RAW264.7, a murine macrophage cell line, was from the American Type Culture Collection TIB-71 (ATCC, Manassas, VA). B299 was reported as a fetal embryonic cell line described earlier (22).

Patient ascites and their corresponding PDX models are listed in **Table 1**. Patient-derived malignant cells from the ascites were subcutaneously injected into nude or SCID mice (4–6 weeks old) with 2 × 10^6^ cells at back flanks of the mice. Tumor progression was monitored and measured by a digital caliber weekly, tumor volume (TV) was calculated as TV = (width^2^ × length)/2. When tumors were too big (close to 20 mm in diameter), or an experiment terminated, mice were sacrificed, and tumors were excised. Tumor tissues were flash-frozen onto dry ice and preserved in -80°C freezer for later use and some were fixed in 10% formalin for immunohistochemical staining. To establish a patient-derived tumor cell line from PDX, portions of fresh tumors were minced into fine pierces with sterile blades and cultured into plates in regular growth medium.

**Table 1:** Patient ascites of gastric cancers and their corresponding PDX tumors used in this study.

Patient ascites IP number	GA PDX number	SITE Siewert class	Tumor Grade	Histological Type of Gastric Cancer	Race	Sex
IP-004	GA030216	Gastric	G3 Poorly differentiated	Diffuse	Hispanic	Male
IP-020	GA062216	AEG II	G2 Moderately differentiated	Intestinal	Hispanic	Male
IP-039	GA090916	AEG II	G2 Moderately differentiated	Intestinal	White	Male
IP-026	GA072516	Gastric	G3 Poorly differentiated	Diffuse	White	Male
IP-054	GA110216	AEG II	G3 Poorly differentiated	Diffuse	White	Male
IP-068	GA011017	AEG II	G3 Poorly differentiated	Diffuse	White	Female
IP-116	GA082517	AEG I	G3 Poorly differentiated	Diffuse	White	Male
IP-107	GA072117	Gastric	G3 Poorly differentiated	Diffuse	Hispanic	Female
IP-013	GA051816	Gastric	G3 Poorly differentiated	Diffuse	Black	Female

### Total RNA extraction, first-strand cDNA synthesis, and mRNA expression qPCR quantification

For mRNA expressions using qPCR, total RNAs were extracted by using the Trizol (Ambion, Austin, TX) method. Cells growing in plates were washed with PBS, then 1 ml Trizol in a 10-cm plate was used for total RNA extraction following the manufacturer’s protocol. Total RNA concentrations were measured on a Nanodrop 1000 machine. First-strand cDNAs were synthesized using the New England BioLabs (NEB, Ipswich, MA) LunaScript RT Supermix kit (#E3010).

qPCRs were performed on an Applied Biosystems (Carlsbad, CA) Quantistudio3 machine. For mRNA expression, first-strand cDNAs were diluted 10 times and a 2.5 µl sample was added to a 10 µl qPCR reaction, which was mixed with 5 µl of 2x PowerUp SYBR Green Master mix (#A25742), 0.1 µl of specific paired primers. For relative quantification of mRNA expression, the classical formula is RQ = 2^(-ΔΔCt), where RQ stands for relative quantification, relative expression fold changes were calculated.

### Genomic DNA extraction, intronic genomic qPCR primer design, and qPCR quantification

Gapdh is one of the most commonly used housekeeping genes for gene expression comparisons (23–25). For genomic copy number quantification, qPCR primers were designed for human Gapdh (hGapdh, NC_000012.12) and mouse Gapdh (mGapdh, NC_000072.7) in their intron regions flanking short exons of both genes in a such way that qPCRs only amplify unique genomic copies, but not mRNA-expressed cDNA copies. The hGapdh primer set detects human genomic copies only and the mGapdh primer set detects murine genomic copies only. Short introns flanking exons of Gapdh make them unique in the whole genomes, regardless of whether the hGapdh and mGapdh exons express the same amino acid sequences in the middle. Primers fully annealing to the genomic sequences determine the initiation of PCRs, also regardless of the amplicons’ internal sequences.

The genomic hGapdh was designed with a primer set of hGapdh.int3.F and hGapdh.int4.R and an amplicon of 189bp; the genomic mGapdh was designed with a primer set of mGapdh.int1.F and mGapdh.int2.R and an amplicon of 260bp. The TaqMan probes for hGapdh and mGapdh were designed for TaqMan qPCRs (**Table 2**). Intronic qPCR products were visualized in 1.2% agarose gels in 1× TAE (Tris-acetate-EDTA) Buffer.

**Table 2:** Primers used in this study.

Primers	Species (Human or Mouse)	Genomic or mRNAs	Sequences
hGapdh.Int3.F	Human	Genomic	5’ CTGACTCAGCCCTGCAAAG 3’ (on Intron3)
hGapdh.Int4.R	Human	Genomic	5’ CCTGCCTTCCTCACCTGAT 3’ (on Intron4)
hGapdh-Taqman-probe	Human	Genomic	5’ FAM-ACTGTCTGCTTCTCTGCTGTAGGCTCA-TAMRA 3
mGapdh.Int1.F	Mouse	Genomic	5’ GGCCACGCTAATCTCATTTT 3’ (on Intron1)
mGapdh.Int2.R	Mouse	Genomic	5’ AAGGCGGAGTTACCAGAGGT 3’ (on Intron2)
mGapdh-Taqman-probe	Mouse	Genomic	5’ FAM-CGAGCCATCGCCAGGTCCGAGC-TAMRA 3’
mGAPDH.mRNA.F2	Mouse	mRNA	5’ TGGCCTTCCGTGTTCCTAC 3’
mGAPDH.mRNA.R2	Mouse	mRNA	5’ GAGTTGCTGTTGAAGTCGCA 3’
mAldh1a2.mRNA.F	Mouse	mRNA	5’ AGGTGGATATAGACAAGGCAGT 3’
mAldh1a2.mRNA.R	Mouse	mRNA	5’ CCGCCATTTAGGGATTCCATAG 3’
mAkt1.mRNA.F	Mouse	mRNA	5’ CCTTTATTGGCTACAAGGAACGG 3’
mAkt1.mRNA.R	Mouse	mRNA	5’ GAAGGTGCGCTCAATGACTG 3’
mACTA2.mRNA.F	Mouse	mRNA	5’ GGCACCACTGAACCCTAAGG 3’
mACTA2.mRNA.R	Mouse	mRNA	5’ ACAATACCAGTTGTACGTCCAGA 3’
mCD44.mRNA.F	Mouse	mRNA	5’ TCGATTTGAATGTAACCTGCCG 3’
mCD44.mRNA.R	Mouse	mRNA	5’ CAGTCCGGGAGATACTGTAGC 3’
mCD133.mRNA.F	Mouse	mRNA	5’ ACTGAGAAATCCCCTACTGAAGT 3’
mCD133.mRNA.R	Mouse	mRNA	5’ GGCCTGTTTCGGCTTTCCTT 3’
mKRT19.mRNA.F	Mouse	mRNA	5’ GGGGGTTCAGTACGCATTGG 3’
mKRT19.mRNA.R	Mouse	mRNA	5’ GAGGACGAGGTCACGAAGC 3’
mCol1A1.mRNA.F1	Mouse	mRNA	5’ CTGGCGGTTCAGGTCCAAT 3’
mCol1A1.mRNA.R1	Mouse	mRNA	5’ TTCCAGGCAATCCACGAGC 3’
mCol1A1.mRNA.F2	Mouse	mRNA	5’ TAAGGGTCCCCAATGGTGAGA 3’
mCol1A1.mRNA.R2	Mouse	mRNA	5’ GGGTCCCTCGACTCCTACAT 3’
mCXCL12.mRNA.F	Mouse	mRNA	5’ TGCATCAGTGACGGTAAACCA 3’
mCXCL12.mRNA.R	Mouse	mRNA	5’ TTCTTCAGCCGTGCAACAATC 3’
mCXCR4.mRNA.F	Mouse	mRNA	5’ CTTCTGGGCAGTTGATGCCAT 3’
mCXCR4.mRNA.R	Mouse	mRNA	5’ CTGTTGGTGGCGTGGACAAT 3’
mEGFR.mRNA.F	Mouse	mRNA	5’ GCCATCTGGGCCAAAGATACC 3’
mEGFR.mRNA.R	Mouse	mRNA	5’ GTCTTCGCATGAATAGGCCAAT 3’
mEpcam.mRNA.F	Mouse	mRNA	5’ CTGGCGTCTAAATGCTTGGC 3’
mEpcam.mRNA.R	Mouse	mRNA	5’ CCTTGTCGGTTCTTCGGACTC 3’
mKRAS.mRNA.F	Mouse	mRNA	5’ GCAAGAGCGCCTTGACGATA 3’
mKRAS.mRNA.R	Mouse	mRNA	5’ GTCCCTCATTGCACTGTACTC 3’
mMTor.mRNA.F	Mouse	mRNA	5’ CAGTTCGCCAGTGGACTGAAG 3’
mMTor.mRNA.R	Mouse	mRNA	5’ GCTGGTCATAGAAGCGAGTAGAC 3’
mHRAS.mRNA.F	Mouse	mRNA	5’ TTTGTGGACGAGTATGATCCCA 3’
mHRAS.mRNA.R	Mouse	mRNA	5’ TGCTCCCTGTACTGATGGATG 3’
mTrp53.mRNA.F	Mouse	mRNA	5’ AGCCAGACGGATCTTGTGAG 3’
mTrp53.mRNA.R	Mouse	mRNA	5’ TCCCGCACAGCAAACTTTATC 3’
mRB1.mRNA.F	Mouse	mRNA	5’ CAAATTAGAACGGACGTGTGAAC 3’
mRB1.mRNA.R	Mouse	mRNA	5’ ACCAGGTCATCTTCCATCTGT 3’
mSox9.mRNA.F	Mouse	mRNA	5’ CGGGGCTGGTACTTGTAATC 3’
mSox9.mRNA.R	Mouse	mRNA	5’ GAGCTCAGCAAGACTCTGGG 3’
mYap1.mRNA.F	Mouse	mRNA	5’ AGGCAGAATTCATCAGCGTC 3’
mYap1.mRNA.R	Mouse	mRNA	5’ TGGCCAAGACATCTTCTGGT 3’
mVEGFA.mRNA.F	Mouse	mRNA	5’ GCACATAGAGAGAATGAGCTTCC 3’
mVEGFA.mRNA.R	Mouse	mRNA	5’ CTCCGCTCTGAACAAGGCT 3’

For genomic DNA extraction from cell lines growing on plates, tumors, and patient samples, the Monarch genomic DNA purification kit from New England Biolabs (NEB, #T3010) was used. For quantifying genomic DNA (gDNA) copies, 1 µl gDNAs was added to a 10 µl qPCR reaction of 2x master mixes. To compare the TaqMan method to SYBR Green, only the TaqMan Master mix (#4444557) was used instead of SYBR Green. All the primers used in this study are listed on **Table 2**.

### Calculations of percentages and copy numbers of human and murine genomic DNAs in PDX tumors and cell lines, and sensitivity of intronic qPCR detection

Genomic DNA copy number calculation by qPCR is based on the principle that a twofold increment of amplicons is amplified over 40 cycles, and on the condition that two amplicons are amplified with equal efficiency. The principle of relative quantification RQ = 2^-ΔΔCt^ was applied as well, since there is no control or a comparator for a specific sample to compare to human hGapdh copies *versus* murine mGapdh copies within a given sample, and *vice versa*, which are values of ΔCt = hCt-mCt, or mCt-hCt, and thus the Ct value difference (-ΔCt) translating into relative copy numbers within a specific sample is sufficient to calculate folds of hCt/mCt or mCt/hCt copies. Mathematically, 2^6.64386 = 100; 2^9.96578 = 1,000; 2^13.2877 = 10,000. For example, if -ΔCt value reaches 6.64386, there would be 100 folds of hCt/mCt or mCt/hCt copies; if -ΔCt value reaches 9.96578, there would be 1,000 folds of hCt/mCt or mCt/hCt copies; if -ΔCt value reaches 13.2877, there would be 10,000 folds of hCt/mCt or mCt/hCt copies. Undetected Ct readings at the 40^th^ cycle are recorded as 40.

Human and murine percentages are calculated as the following, and *hP* + *mP*=100%.


hP= folds of human hGapdh/(folds of human hGapdh+one fold of mouse mGapdh)×100%

For converting human or murine genomic DNA weights into genomic DNA copies, this formula is used:


$$\text{DNA copy numbers}=(\text{DNA amount}\times 6.022\times 10^{23})/(\text{length}\times 1\times 10^{9}\times 650)$$

where amount is the DNA weight in ng, the length for human genome is 3.2 billion nucleotides, and the length for mouse genome is 2.5 billion nucleotides.

Cell percentage, genomic copy numbers, means, and SEM (standard errors of the means) were calculated in triplicates, error bars were added on all bar graphs; even some are so minimal.

The sensitivity of detecting genomic copies by intronic qPCR was estimated based on the same principle elaborated as above: readings of ΔCt = hCt-mCt or mCt-hCt were interpreted as sensitivity = 1% if ΔCt = 2^6.64386 = 100; sensitivity = 0.1% if ΔCt = 2^9.96578 = 1,000; and sensitivity = 0.01% if ΔCt = 2^13.2877 = 10,000.

### Calculation of cell line doubling time

For cells growing on plates, the doubling time was calculated using the following formula:

Doubling Time dt = {T×(ln2)}/{ln(Xe/Xb)}, where T = time units; Xb = starting cell numbers; Xe = ending cell numbers.

### Statistical analyses

Data were analyzed using the Student *t*-test; the significance of differences between groups was judged using a two-tailed *t*-test. Results were statistically significant when p value was<0.05, and highly significant when p value was<0.01. Graphs were created either with Microsoft Excel or GraphPad Prism. qPCRs adopted three replicates for each sample, each qPCR experiment was repeated at least one more time. Bar graphs, vertical and horizontal, were plotted as means ± SEMs; even SEMs were too minuscule on some graphs. PDX tumor sizes from five mice of each treatment of *in vivo* studies were analyzed in the same fashion and plotted in GraphPad Prism.

### Lentiviral mCherry-Luciferase labeling of cell lines

The main passaged cells P0825 was labeled with lentiviral mChLuc, generated from a lentiviral plasmid FUW-CBRLuc-mCherry, a gift from Dr. Helen Piwnica-Worms, M.D Anderson Cancer Center. Lentiviral production with pCMV-Dr8.2 and pCMV-VSV.G in HEK293T cells and target cell transduction were described earlier (20). *In vivo* bioluminescent images (BLI) were taken with intraperitoneal injection of 150 μl 15 μg/μl D-Luciferin in mice and analyzed by Perkin Xenogen IVIS 200 Imaging System (PerkinElmer, Waltham, MA).

### G-banded karyotyping analysis and STR fingerprinting

The G-banded karyotyping analysis of human H0825 cells and main passaged murine P0825 cells descended from the GA082517-PDX was performed by our institutional Cytogenetics and Cell Authentication Core (CCAC) in the Department of Genetics at MD Anderson Cancer Center, and was described earlier (26).

STR fingerprinting analysis of genomic P0825 (06/23/21) was performed by ATCC, the genomic DNA was extracted from the same batch of cells as P0825, M0825, and H0825 used for the *in vivo* studies of PDX models on 06/23/2021.

### Immunofluorescence

Pertinent cell lines were growing in 12-well plates with sterile cover slips immersed at bottom when cells were seeded. IF was performed as previously described (27). Antigen retrieval was performed using Antigen Unmarking Solution (BioGenex Laboratories, Cat# HK0805K-GP). Antibodies against the following markers were: KRT19 (CK19) (AbboMax Cat# 602-670, 1:100), EpCam (Cell Signaling, Cat# 2929S, 1:100), FAP (Invitrogen, Cat# PA5-99313, 1:100), Sox9 (EMD Millipore, Cat# AB5535, 1:100), Yap1 (Abcam, Cat# ab205270, 1:100), Vimentin (Santa Cruz, Cat# SC6260, 1:100). The staining was followed by secondary antibody Alexa Fluor 488 (donkey anti-mouse, Invitrogen, Cat# A21202, 1:500) or Alexa Fluor 555 (donkey anti-rabbit, Invitrogen, Cat# A31572, 1:500). Slides were then mounted with DAPI-containing Vecta Shield Mounting Medium (Vector Laboratories, Cat# H-1200-10) and visualized under a Nikon T2 confocal laser scanning microscope. The scale bar marks 50 µm in IF images.

### Whole exome sequencing analyses

For WES analysis, the human samples were: 1) patient IP-116 blood; 2) patient ascites IP-116; 3) GA0825-PDX-G1 tumor from IP-116 in a nude mouse; 4) human H0825 cells growing on plates; and 5) H0825-PDX tumor (#978R) in a SCID mouse. The murine samples were: 1) normal blood from a SCID mouse, 2) murine P0825 cells growing on plates, 3) P0825 PDX tumor (#984L) in a SCID mouse, and 4) murine M0825 cells growing on plates. WES capture of samples and libraries are listed in **Supplementary Table S1**. Genomic DNAs extracted from patient ascites, corresponding PDXs and cell lines were submitted to our institutional Sequencing and Microarray Core Facility at MD Anderson Cancer Center for WES capture. For data processing, genotyping quality check, genomic mutation calling, filtering, functional annotation, and DNA copy number analysis, etc., see Wang et al. (2021) (28).

## Results

### Intronic qPCR efficiently quantified hGapdh and mGapdh copies fast yet with high sensitivity

**Figure 1:**
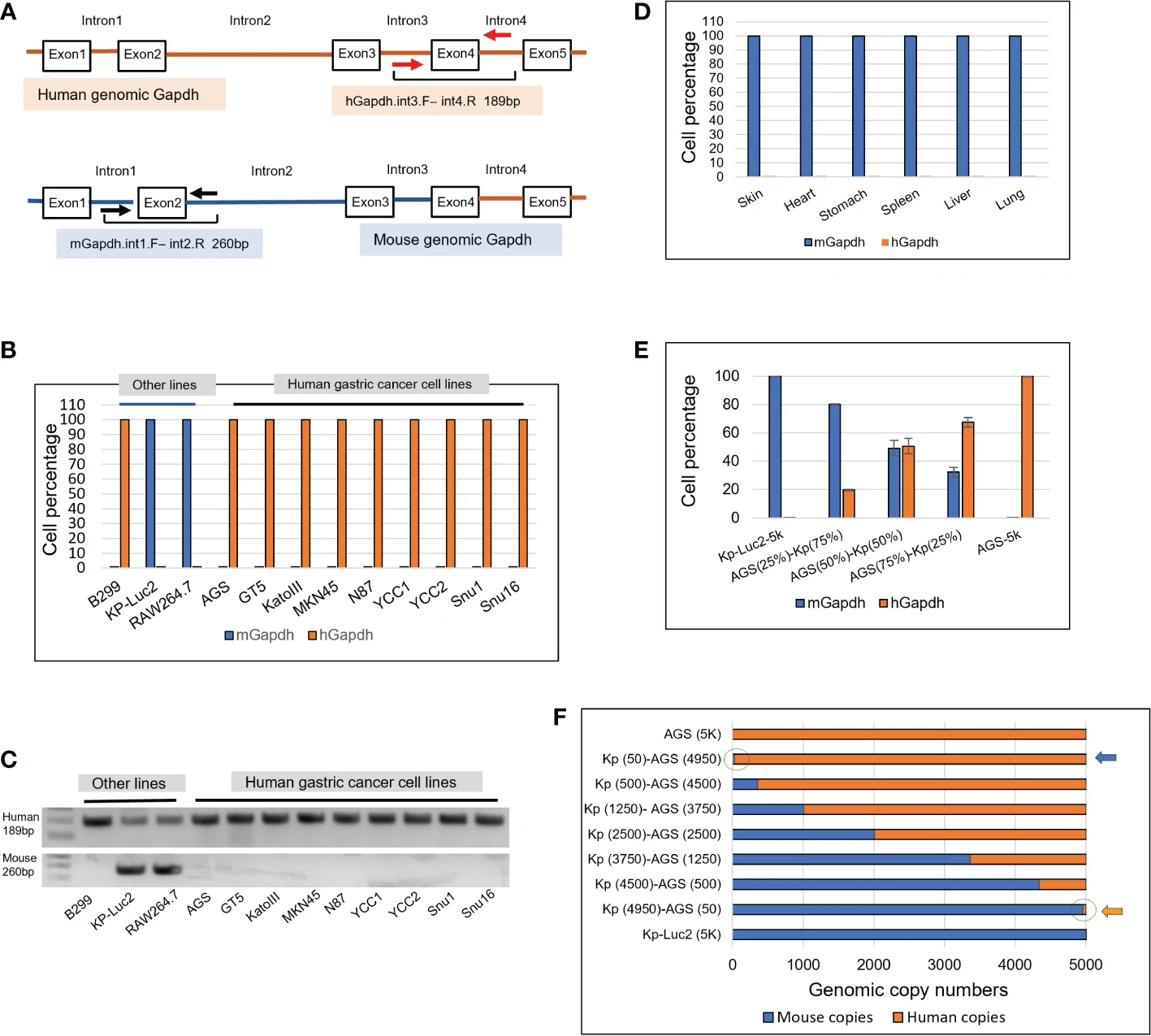
Design of intronic qPCR primers on genomic Gapdh gene and its quantification on human and murine genomic Gapdh copies as well as tests of amplification efficacy and sensitivity. **(A)** A schematic diagram of the genomic human and murine Gapdh gene (hGapdh and mGapdh) shows the locations of two sets of intronic qPCR primers. **(B)** Intronic qPCR method was applied to authenticate gastric cancer and other cell lines. **(C)** qPCR end products corresponding to B were visualized on agarose gels, showing human amplicon 189bp and murine 260bp. **(D)** Intronic qPCR quantification was tested on organs of a SCID mouse. **(E)** Efficiency test of qPCR quantification of hGapdh and mGapdh in different combinations of copy numbers, using human cell line AGS and murine line Kp-Luc2. **(F)** Sensitivity test of qPCR quantification of copy numbers using the same two lines as in E, arrows pointed to the circles where 50 copies of human genome and murine genome were visualized.

A genomic scheme is shown for the hGapdh with primers hGapdh.int3.F and int4.R anchored on introns 3 and 4 flanking the exon4, and the mGapdh with primers mGapdh.int1.Fand int2.R anchored on introns 1 and 2 flanking exon2 (**Figure 1A**). Intronic qPCRs detect genomic Gapdh at one copy per cell, but not Gapdh mRNAs-transcribed cDNAs. Both sets of primers successfully amplified in human and murine cells (**Figures 1B–F**).

As shown in **Figure 1B**, intronic qPCR quantifying hGapdh and mGapdh copies verified that all the nine gastric cancer lines AGS, GT5, KatoIII, MKN45, N87, YCC1, YCC2, Snu1, and Snu16 were purely human. For the other three cell lines, B299 was a purely human line, RAW264.7 and Kp-Luc2 were both purely murine (**Figure 1B**). Their qPCR end products corresponding to the samples in **Figure 1B** were visualized in agarose gels (**Figure 1C**), which showed that mGapdh amplification was more stringent than hGapdh. hGapdh end products may be attributed to the Taq in the ABI SYBR Green master mix is a non-high-fidelity polymerase that could ramp up non-specific amplifications, even though one hGapdh primer anneals only to the human intronic genome (**Figures S1, S2**), but the qPCR ΔCt differences were big enough to dismiss any non-specific amplification. The protein sequence alignment of human Gapdh and mouse Gapdh is presented in **Figure S3**, showing a very similar amino acid sequence with an identity of 87.5%. To further validate our primers’ specificity, six murine organs were harvested from a SCID mouse, genomic DNAs were extracted, and intronic qPCR verified that they all were composed of purely murine cells (**Figure 1D**).

To test if the hGapdh amplicon and the mGapdh amplicon were amplified in qPCR reactions with equal efficiencies, 5000 genomic copies of AGS (human), 5000 genomic copies of Kp-Luc2 (mouse), and mixed populations of both at different ratios (75%/25%; 50%/50%; and 25%/75%, respectively) were put into qPCR quantification. The result showed that detection of both hGapdh and mGapdh amplicons was equally efficient, as shown in **Figure 1E**, both hGapdh and mGapdh copy numbers showed a linear decrement and increment, respectively. The slight deviation might be attributed to pipetting error because in every reaction of 10 µl volume, only 1 µl genomic DNA was manually added. To further test if genomic DNA qPCR could be sensitive enough to detect 50 copies of genomic DNA in a 10 µl reaction in a percentile graph (100% = 5000 copies), 5000 copies of AGS (human) and 5000 copies of Kp-Luc2 (mouse) genomes were mixed at different copy number proportions, the fewest copies were 50 and qPCR was able to easily pick up the 50 copies in a copy number view in X-axis (blue and orange arrows pointing to cycles) (**Figure 1F**). A same setup qPCR as in **Figure 1F** but using TaqMan method and two probes is presented in **Supplementary Figure S4**, and a comparison between SYBR Green qPCR and TaqMan qPCR is presented in the discussion section.

**Figure 2:**
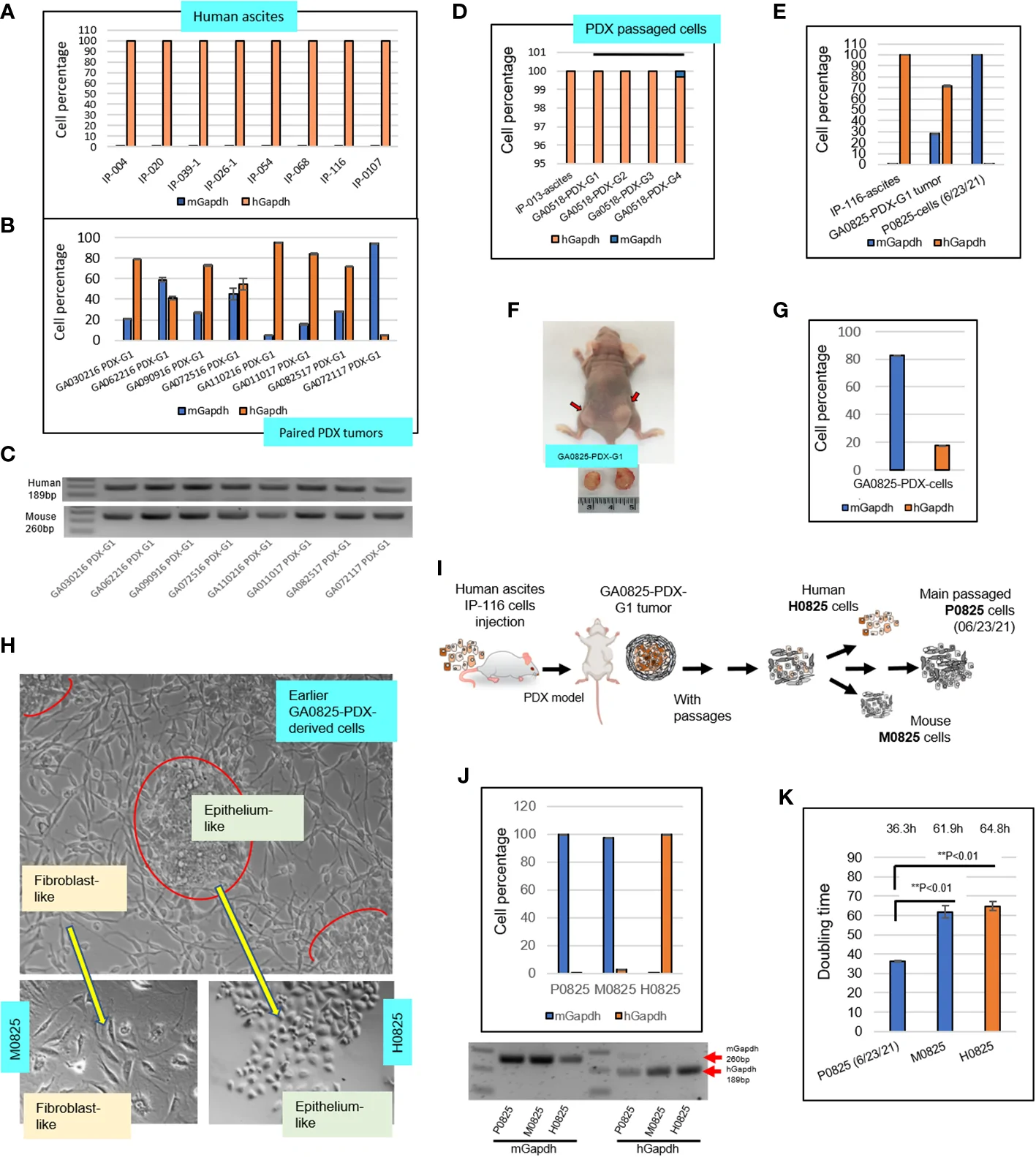
Intronic genomic qPCR quantification of human ascites and their paired PDX tumors, and further investigation on transformation of a PDX model GA082517-PDX-G1. **(A)** Intronic qPCR quantified compositions of eight human ascites. **(B)** Intronic qPCR quantified compositions of the PDX tumors paired to the eight ascites in A, showing different levels human and murine cell percentages. **(C)** qPCR end products were visualized on agarose gels, showing human amplicon 189bp and murine 260bp. **(D)** qPCR quantification of composition in four passaged cells of GA051816-PDX-G1, -G2, -G3 and -G4 in SCID mice. **(E)** Intronic qPCR quantification was applied to a special case of GA082517-PDX-G1, its composition completely turned over from the human ascites IP-116 to PDX and to its derived cells P0825 (6/23/2021). **(F)** Human gastric cancer patient IP-116 ascites were injected into a nude mouse to generate GA082517-PDX-G1 at time of sacrifice. **(G)** Intronic qPCR quantified cell composition of an *in vitro* growing cells derived from GA0825-PDX-G1. **(H)** Micrographs of the cells in G showed that there were two types of morphologically different cells, from which single-cell pools of epithelium-like H0825 and fibroblast-like M0825 were isolated. **(I)** An illustration of the GA0825-PDX-G1-derived cells showed how the lineage diverged into H0825, M0825 and the cells passed on to main passaged P0825 (6/23/21). **(J)** Intronic qPCR quantified the compositions of three subpopulations human H0825, murine P0825 and M0825, their PCR end products were visualized on an agarose gel. **(K)** Doubling times were measured for three subpopulations human H0825, murine P0825, and M0825 cells.

In our experiments, this intronic genomic qPCR was very sensitive to pick up a few copies during our many runs. Among 12 human/murine cell lines that were put into the qPCR run (**Figure 1B**), Kp-Luc2 was with the lowest Ct value difference, ΔCt = 11.48, since 2^11.48 = 3.5 × 10^4^, which also translated into<3.5 × 10^-4^ probability that there was human cell contamination in this cell line. Another example would be seen in **Figure 2D**, where there were 0.303% murine cells detected in the 4^th^ PDX-passage GA0518-PDX-G4. As for other GA0518-PDX-G1, -G2, and -G3 cells in **Figure 2D**, they were detected with Ct value differences -ΔCt >20 (hGapdh/mGapdh), i.e. >2^20 = 1,048,576 folds of hGapdh copies compared to mGapdh copies, which indicated there is a<10^-6^ probability that there were murine cells.

The above analysis verified that this intronic genomic qPCR method is more sensitive than the aforementioned 95% accuracy of fluorescence-activated cell sorting (FACS) and ssPAL analysis (10), 5%–10% sensitivity of STR and 3%–5% sensitivity of SNP assays, and its sensitivity is on par with or better than the 0.1% sensitivity level of the NGS method (11).

### PDXs carried over murine cells and GA0825-PDX-G1 transformed into a murine line

Eight ascitic samples from gastric cancer patients were selected and tested with intronic hGapdh and mGapdh qPCR, and the result showed that all eight samples were composed of purely human cells (**Figure 2A**); while the human PDX tumors paired to the eight patients’ ascites harvested from mice all carried different levels of murine stromal cells. Sample GA110216 PDX-G1 had a mix of 95.28% human and 4.72% mouse cells; while GA072117 PDX-G1 had only 5.36% human cells and 94.64% murine cells (**Figure 2B**). The latter PDX tumor presented an obvious dilemma for *in vivo* drug treatment studies and preclinical personalized therapies if chosen for experiments. The corresponding qPCR end products of **Figure 2B** were visualized on an agarose gel (**Figure 2C**). Patient-derived gastric cancer line GA0518 derived from the PDX model of human ascites IP-013 has become an important cell line for gastric cancer research in our lab (26). In intronic genomic qPCR on patient ascites IP-103 and its derived GA0518-PDX cells after 1^st^, 2^nd^, 3^rd^, and 4^th^ generation of PDX xenografts, only the cells from the 4^th^ generation of PDX tumor had a small fraction of murine cells, a 0.303% of total cells (**Figure 2D**).

Another particular PDX sample GA082517-PDX-G1 (**Figure 2F**) had a mix of 71.92% human cells and 28.08% murine cells, from which murine cells gradually dominated and finally became purely murine over passages (**Figures 2E, F**). **Figure 2E** depicted a radical turnover from a purely human patient IP-116 ascites, to a mixed human/murine GA082517-PDX-G1 tumor, and then to a complete murine cell line P0825. P0825 had a ΔCt (mGapdh vs. Gapdh) = 16.747, i.e. a murine line with substantial confidence based on qPCR ΔCt value difference. **Figure 2F** showed this GA0825-PDX-G1 model generated from patient ascites IP-116 in a nude mouse. An earlier GA082517-PDX-G1-derived cell population growing *in vitro* (**Figure 2H**), had a mixed 17.45% human/82.6% murine cells (**Figure 2G**). From this population (**Figure 2H**, top photo), single-cell cloning was performed in 96-well plates, 5–10 cells with similar morphology were pooled together and dubbed epithelium-like H0825 and fibroblast-like M0825, indicating human and mouse respectively (lower photos, **Figure 2H**). Intronic genomic qPCR determined they were human for H0825 and murine for M0825 (**Figure 2J**). The cell line established by many passages (≥15 passages) from the earlier GA082517-PDX-G1-derived cells was named P0825 (from the date 06/23/2021 on). Agarose gelling of intronic genomic qPCR end products for P0825, M0825, and H0825 confirmed that P0825 and M0825 were purely murine and H0825 was human (**Figure 2J** bottom photo). **Figure 2I** illustrates how H0825, P0825, and M0825 diverged from the common ancestral GA0825-PDX-G1 cells.

Doubling times (**Figure 2K**) for the three subpopulations were P0825: 36.3 h, H0825: 61.9 h, and M0825: 64.8, confirming that P0825 propagated much faster than the other two. The doubling time for a cell line is important, as the fast-growing clones would dominate and overtake other clones in short time.

### P0825, H0825, and M0825 displayed different tumorigenic capability

**Figure 3:**
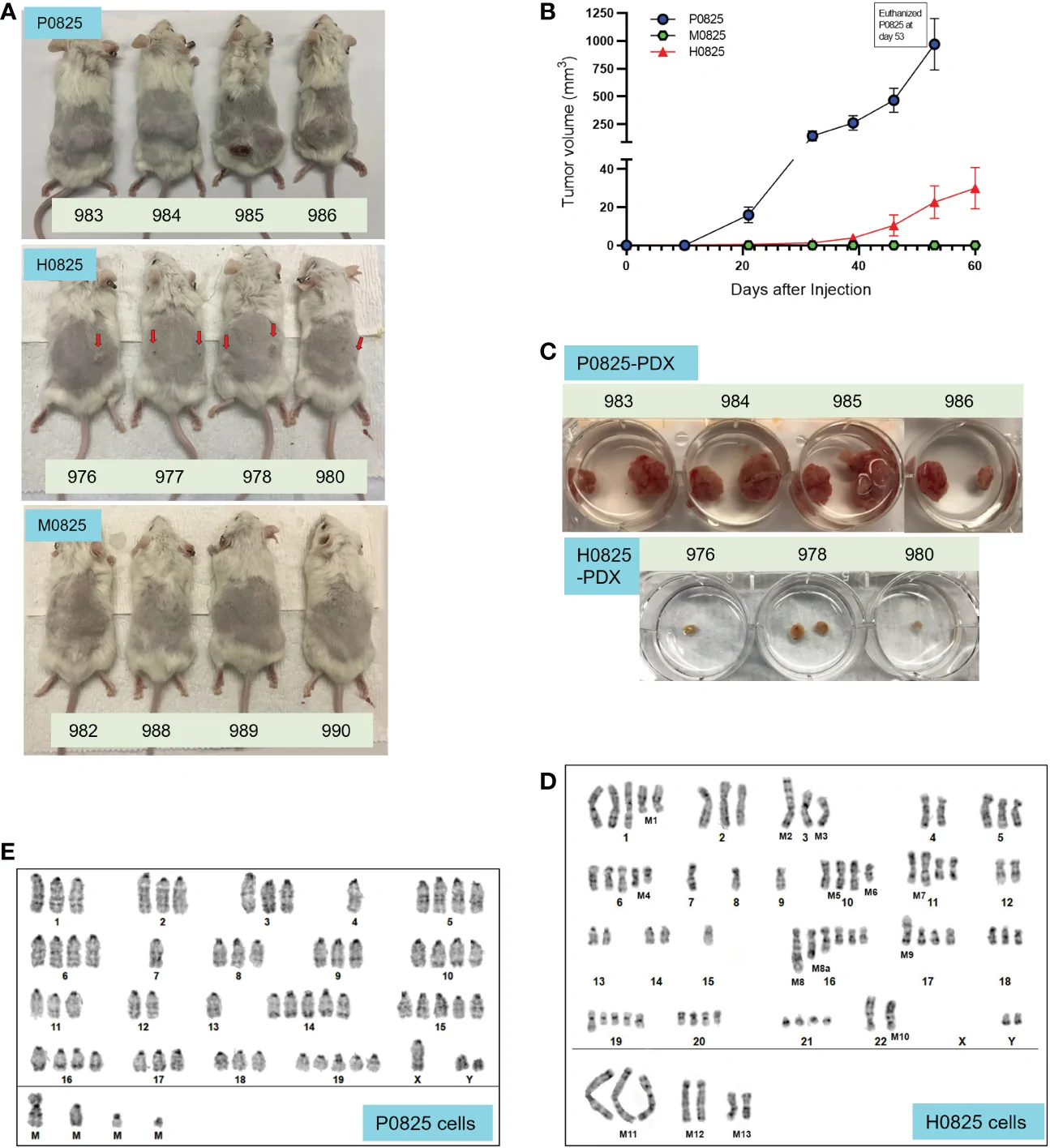
From a common GA082516-PDX-G1, three descendent subpopulations generated PDX models, their tumor size progression and G-banded karyotyping analyses of P0825 and H0825 cells. **(A)** Murine P0825, human H0825, and murine M0825 cells were injected into SCID mice subcutaneously to generate PDX models. Red arrows in H0825 pointed to the tiny bumps of possible tumors. **(B)** Time progression of tumor sizes in mice generated by human H0825, murine P0825, and M0825 cells. **(C)** Tumors excised from PDX mice at time of sacrifice showing tumors from murine P0825- and human H0825-generated PDXs. **(D)** G-banded karyotyping analysis of human H0825 cells. **(E)** G-banded karyotyping analysis of murine P0825 cells.

Derived from the common ancestral GA082517-PDX-G1 cells, equal numbers of 2 × 10^6^ cells from the three subpopulations P0825, H0825, and M0825 were injected subcutaneously into two back flanks of SCID mice, which displayed differences in tumorigenicity. P0825 was aggressively tumorigenic (**Figure 3A** top photo, **Figure 3C**), H0825 was weakly tumorigenic (**Figure 3A** middle photo, **Figure 3C**), and M0825 was not tumorigenic (**Figure 3A** bottom). **Figure 3B** illustrated tumor volumes over time, showing P0825 becoming so prominent in tumorigenicity over the other two subpopulations. Given their different doubling times in **Figure 2K**, it made more sense that human cancer cells H0825 were gradually lost due to slower growth in the mix. H0825 subpopulation was pooled with 5–10 epithelium-like single-cell clones isolated from earlier GA082517-PDX-G1 cells, and there also might be a selection bias leading to its weak tumorigenicity. By the same logic, M0825 subpopulation was combined with 5–10 fibroblast-like single cell clones from the GA082517-PDX-G1 cells, and it suffered the same selection bias and was proven to be not cancer cells at all, but merely fibroblast-like immortalized murine cells (**Figure 3B**).

### G-banded karyotyping analysis confirmed that P0825 and H0825 were murine and human, respectively

Contrary to the earlier expectation, G-banded karyotyping analysis revealed that P0825 cells represented a murine line, derived from a male mouse. The later submitted cell line H0825 was human, derived from a male as well. Clinical data showed that it was from a white male (**Table 1**), whose cells showed substantial aneuploidy, polyploidy, and other chromosomal aberrations in the karyotyping analysis.

Karyotyping in H0825 cells showed substantial genomic aberrations (**Figure 3D**): polyploidy in chromosomes (Chr) Chr1, 2, 3, 5, 6, 10, 11, 16, 17, 18, 19, 20, 21; monoploidy in Chr7, 8, 9, 15; ChrX was missing; there were chromosomal segment deletions in Chr1 [M1del(1q)], Chr6 [M4del(6q) and Chr10 [M6del(10q)]; there were chromosomal segment translocations in Chr3 [M2t(3p):? and M3t(3q):?], Chr10 [M5t(10q;14q)], Chr11 [M7t(11q:13q)], Chr17 [M9t(17p):?] and Chr22 [M10 t(22p+)].

As for P0825 cells, G-banded karyotyping analysis verified that it was a murine line, with 20 pairs of chromosomes and conspicuous centromere tips of each chromosome (**Figure 3E**). No chromosomal indels and translocation were noted; polyploidy was observed all over on Chr1, 2, 3, 5, 6, 8, 9, 10, 11, 14, 15, 16, 17, 18, 19 pairs and ChrY; and monosomy was observed on Chr4 and Chr7. This karyotyping suggested the cells were derived from a male mouse, i.e., the nude mouse in **Figure 2F**.

### Human gastric cancer ascites transformed murine stromal cells into a murine tumorigenic line

**Figure 4:**
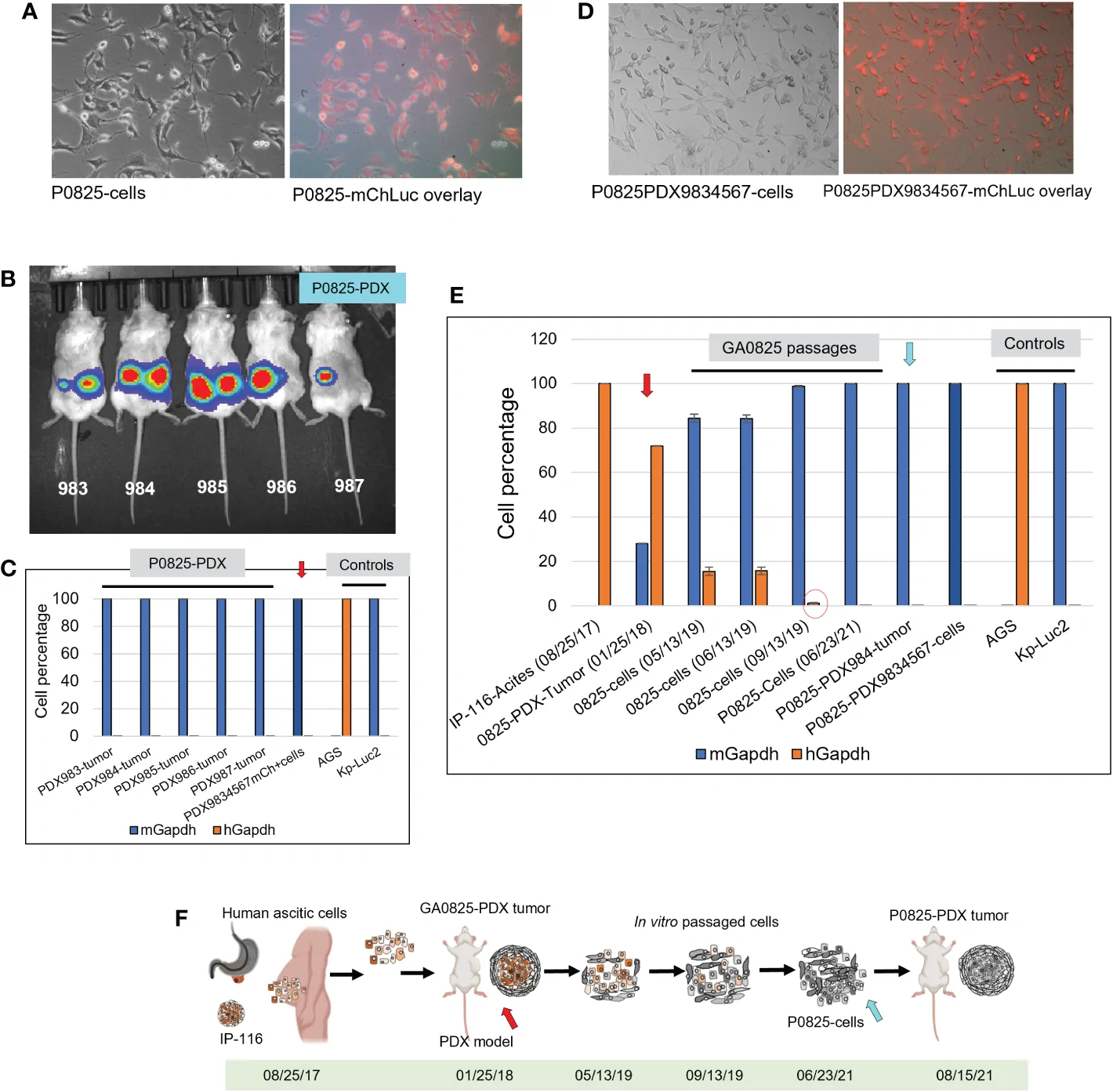
Murine P0825 cells injected into mice to generate PDX models and time progression of human-to-mouse transition and oncogenic transformation. **(A)** P0825 was labeled with lentiviral mCherry (mChLuc), micrographs showed in brightfield and mCherry overlay. **(B)** P0825 cells were injected subcutaneously into five mice to generate PDX models, with luciferase imaging for the mice. **(C)** Intronic qPCR quantification of the PDX tumors from the five mice, with the arrow pointing to the merged cell pool derived from tumors of the five PDX mice. AGS and Kp-Luc2 served as controls for qPCR. **(D)** The merged cell pool P0825PDX9834567 derived from the five PDX tumors from B showed micrographs in brightfield and mCherry overlay. **(E)** Time progression of GA082516-PDX-G1 (with red arrow), its ancestral patient ascites IP-116 and its derived cell pools, intronic qPCR quantified the human-to-mouse transition from patient IP-116 to murine PDX-derived cells over time, showing human percentages decremental and murine percentages incremental. P0825 (06/23/2021) and P0825PDX9834567 cells (with blue arrow) are determined as fully murine cells. **(F)** An illustration depicting time progression of this human-to-mouse transition reflected in E (red and blue arrows matching F with E).

Before injection of P0825, H0825, and M0825 cells into SCID mice to generate the PDX models, the main passaged P0825 was labeled with lentiviral mCherry-Luciferase (mChLuc), and the cells were FACS sorted in our institutional Flow Cytometry Core (**Figure 4A**). All the five mice injected with P0825 cells showed strong luciferase activity with exposure of 1 s at the time of 6 weeks of subcutaneous injection (**Figure 4B**). Tumors from the five mice were excised and portions of each mouse were minced and grown in plates at the time of sacrifice, and five PDX-derived cells merged into one line called P0825-PDX9834567 (**Figure 4C** red arrow). Genomic DNAs of tumors from each mouse and their merged cell line P0825-PDX9834567 were extracted and intronic qPCR quantification showed that all five PDX tumors were 100% murine (ranging the lowest 99.97057073% to the highest 99.99968116%), so were the cells P0825-PDX9834567 (murine 99.99421992%) (**Figure 4C**). This result strongly confirmed that it was murine P0825 (06/23/21) cells that turned on tumorigenesis, ruling out the possibility that the tumors were from a few undetected human cells in P0825. Fluorescence microscopy showed that the P0825-PDX9834567 cells were 100% mCherry positive, proving that the PDX-derived and merged cell pool P0825-PDX9834567 descended from the original injected P0825 (**Figure 4D**); second, it also proved that P0825 was a murine line carrying cancer cells and cancer stem cells; third, a 2^nd^ PDX model (murine P0825-PDX-G2, i.e. P0825-PDX9834567 cells) was generated with aggressive tumorigenicity.

Retrospectively, regarding to the human-to-murine cells transition and oncogenic transformation, samples from original patient ascites IP-116 all the way to the newly generated murine cell pool P0825-PDX9834567 were retrieved and genomic DNAs were extracted. Intronic qPCR determined that human percentages quickly decreased in a time progression from 100% IP-116-Acites (08/25/17), 71.92% GA0825-PDX-G1-Tumor (01/25/18) with red arrow, 15.53% GA0825-cells (05/13/19), 15.74% GA0825-cells (06/13/19), to 1.19% GA0825-cells (09/13/19) (**Figure 4E** red circle). The main passaged cell line P0825 (06/23/21) was already 100% of murine at the time of cell injection into mice for PDXs, which was further proven in **Figure 4C**. This progressive transition from human ascites IP-116 to murine cells GA0825-PDX-G1 and P0825-PDX9834567 is illustrated in **Figure 4F**, which pointed to a possible human-to-murine oncogenic transformation in the generation of GA0825-PDX-G1 (01/25/18) (red arrows) to the P0825 (06/23/21) (blue arrow). The mechanism of how this oncogenic transformation happened, though, it needs further interrogations (**Figure 4E, F**).

### P0825 is a *bona fide* and pure murine line with complete depletion of human cells

Even after many intronic qPCR quantification and analyses in **Figures 2E, J**, **4C**, **4E** quantifying that P0825 (06/23/21) had evolved into a murine line, PDX models in **Figures 3A** top, **3B, C** top, and **4B** proving P0825 is an aggressively tumorigenic line, suspicion hinges on that P0825 (06/23/21) may still harbor a few dozens or hundreds of human tumorigenic ascitic cells at much lower than 1% in the percentile scale. In a second scenario, P0825 (06/23/21) was contaminated by a tumorigenic murine cell line during the GA0825-PDX-G1 to P0825 *in vitro* passages. Further scrutiny (**Supplementary Document 1**) of the original intronic qPCR raw data on the same samples P0825, M0825, and H0825 as that xenografted onto SCID mice on 23 June 2021 provided further evidence that P0825 held 99.9898% murine cells, with a 0.0104% probability of human cells, and the latter number could well be the non-specific amplification of the SYBR Green regular Taq. Yet, 2 × 10^6^ cells of H0825 (human 99.9945%) produced very small tumors, and only four minimalist tumors from eight injections of the four SCID mice (**Figures 3A** middle, **3C**); while P0825 (06/23/21) produced huge tumor volumes (**Figure 3A** top and middle, **3B, C**), and the tumors produced by P0825 (06/23/21) (**Figure 4C**) were all murine in five mice by intronic qPCR, further corroborating that P0825 is an aggressively tumorigenic murine line.

To further characterize P0825 (06/23/21), an STR fingerprinting analysis was performed by ATCC (**Supplementary Document 2**), which drew a conclusion that P0825 is a pure murine line without human or other murine line contamination. Its 18-microsatellite pattern is unique and does not match any of the ~400 murine lines profiled in ATCC.

The above evidence proved that P0825 (06/23/21) had evolved into a *bona fide* and pure tumorigenic murine line over passages and had been depleted of any human ascites-derived cells.

### P0825-PDX tumors highly expressed cancer related markers

**Figure 5:**
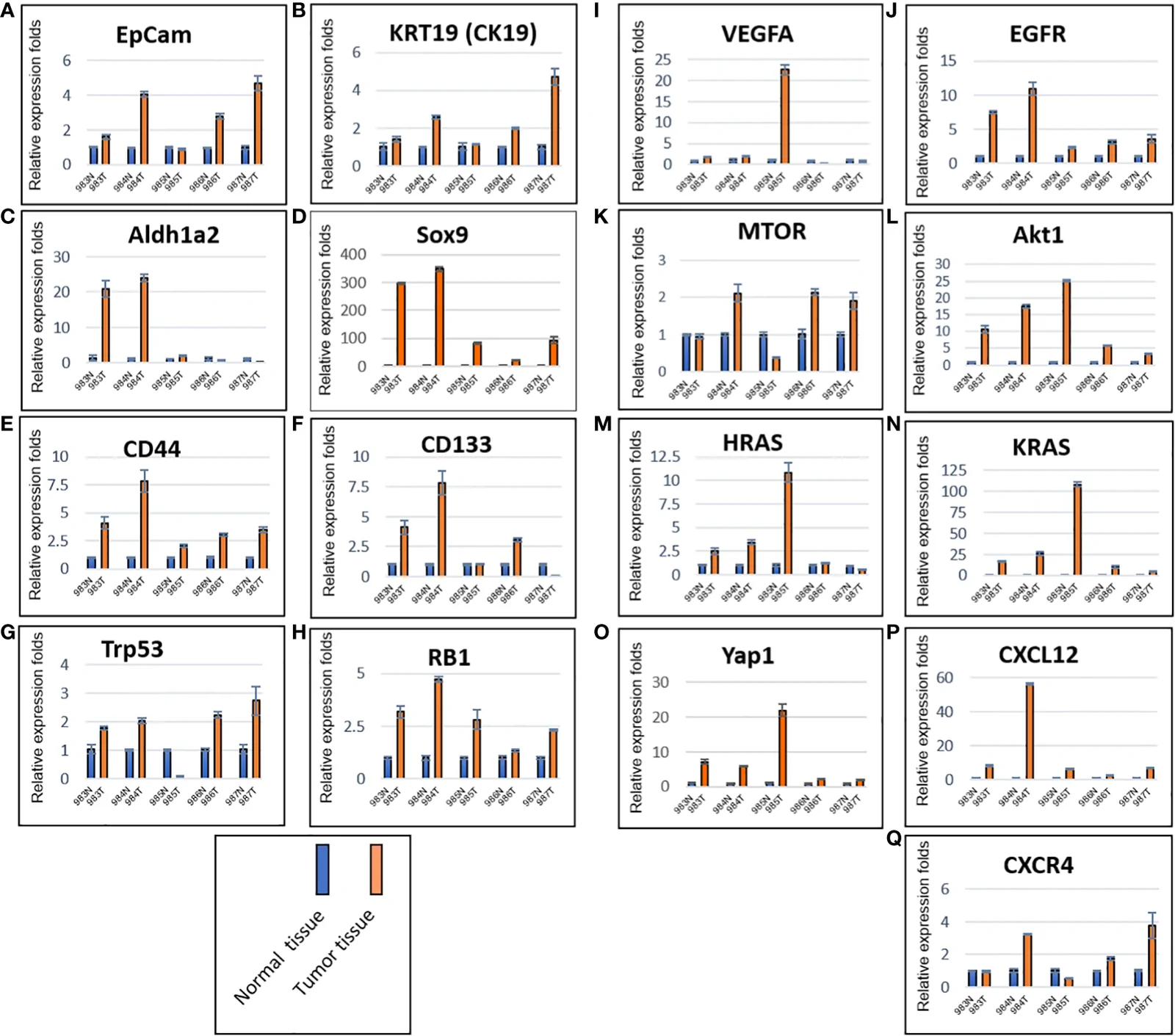
Murine P0825-cells-generated P0825-PDX tumors in SCID mice expressed higher in mRNAs of cancer related markers compared to adjacent normal tissues. Cancer markers EpCam and CK19 **(A, B)**; cancer stem cell markers Aldh1a2, Sox9, CD44, and CD133 **(C–F)**; tumor suppressor markers TP53 and RB **(G, H)**; growth factors VEGFa and EGFR **(I, J)**; oncogenic driver genes mTOR, Akt1, HRas, KRas and Yap1 **(K–O)**; cytokine and chemokine markers CXCL12 and CXCR4 **(P, Q)**.

Total RNAs were extracted from tumors of the five mice (#983, #984, #985, #986 and #987) generated by murine P0825 cells (**Figure 4B**), together from their paired normal skin-muscle tissues 1–2 cm away from each tumor. Gene expression levels were determined for several cancer markers, including EpCam and KRT19 (CK19) (**Figures 5A, B**), notable cancer stem cell markers Aldh1a2, Sox9, CD44, and CD133 (**Figures 5C–F**), tumor suppressor markers Trp53 (TP53) and RB1 (**Figures 5G, H**), growth factors VEGFA and EGFR (**Figures 5I, J**), oncogenic driver genes MTOR, Akt1, HRAS, KRAS and Yap1 (**Figures 5K–O**), and chemokine markers CXCL12 and CXCR4 (**Figures 5P, Q**). EpCam and KRT19 were reported as prognostic cancer markers correlated with clinical outcome (29, 30). All mRNA expressions of the above stated genes were upregulated in most five mice tumors. Higher expression of EpCam and KRT19 in tumor tissues confirmed that they were malignant. Of the stem cell markers, Sox9 expressed much higher in tumors than in normal tissues, and CD44 expressed higher in tumors as well, suggesting cancer stem cells were playing important roles. Of the two tumor suppressors, Trp53 expressed higher in four of five mice tumors than in normal tissues, RB1 expressed higher in all five mice tumors. Of two growth factor-related genes, EGFR expressed higher in all five tumors than normal tissues. Out of five oncogenic driver genes, Akt1, KRAS, and Yap1 stood out higher in all five tumors than normal tissues. Of the chemokine axis CXCL12-CXCR4, CXCL12 expressed higher in all five mice tumors than normal tissues.

Obviously the main passaged P0825, being an aggressively tumorigenic murine line (**Figures 3A** top, **4B**), carried cancer markers and cancer stem cell markers. The tumorigenicity of P0825 stemmed from the patient ascites IP-116 and the GA0825-PDX in a nude mouse (**Figure 2F**), whose stromal cells in the TME were oncogenically transformed by ascites IP-116 as a result of an unknown mechanism. Interestingly, P0825 cells labeled with mChLuc injected into mice did not bring host mice’s stroma cells into the murine line P0825-PDX9834567 since all the cells were mCherry positive (**Figure 4D**).

### Selection bias resulted in M0825’s lack of capacity for tumorigenesis

**Figure 6:**
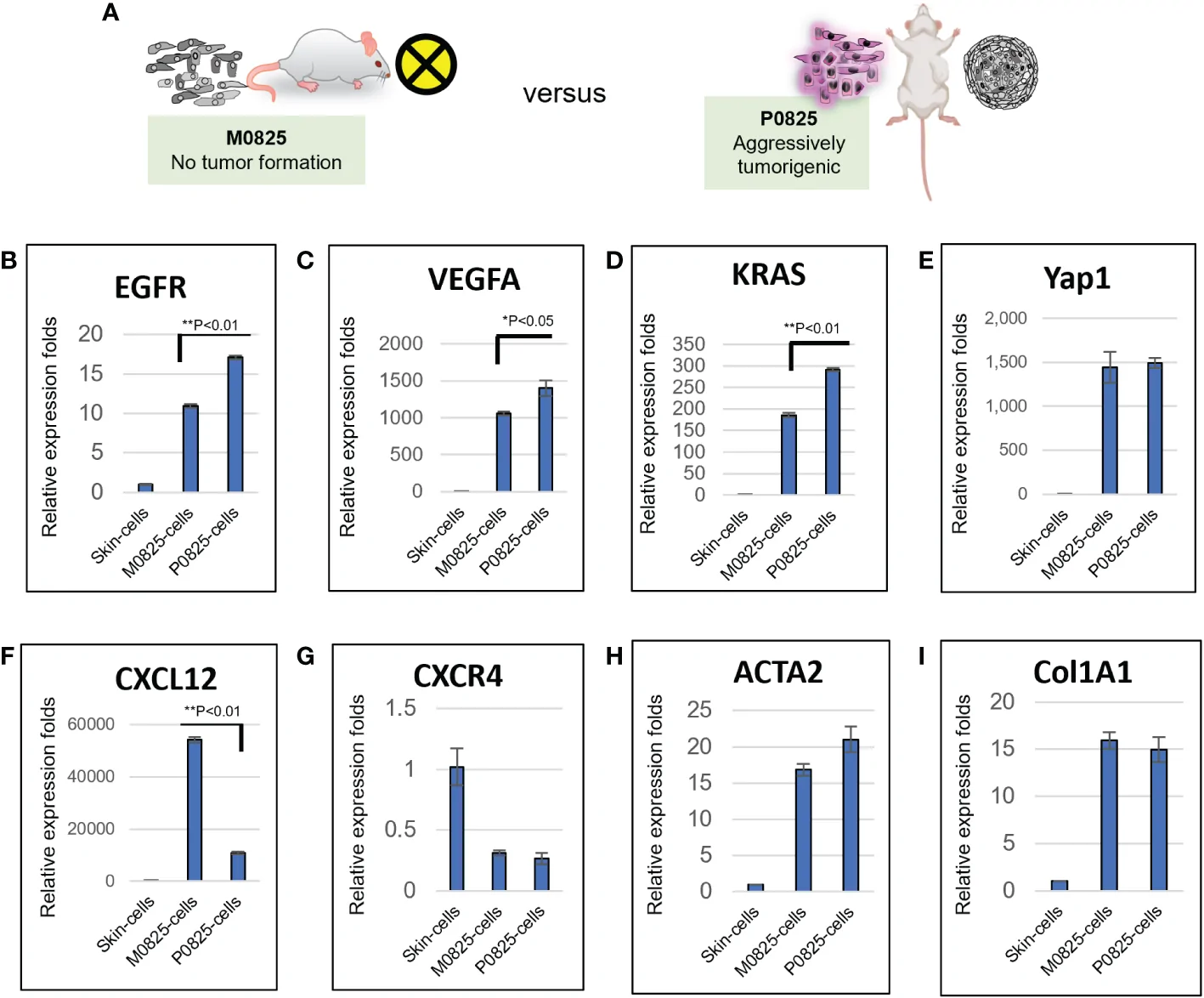
mRNA expression profiling and comparison of M0825 cells, P0825 cells and normal skin cells. **(A)**. An illustration of nontumorigenic M0825 and aggressively tumorigenic P0825. **(B–I)**. mRNA expression comparisons of growth factors VEGFa and EGFR **(B, C)**, oncogenes KRas and Yap1 **(C, D)**, chemokine axis CXCL12-CXCR4 **(F, G)**, CXCL12 is also a fibroblast and immunity marker. mRNA expressions for fibroblast marker α-SMA and Col1A1 **(H, I)**.

M0825 was a pool of 5–10 single cell fibroblast-like clones from the earlier GA0825-PDX-G1 cells, which did not generate tumors in any of the five SCID mice (**Figures 3A** bottom, **3B**, **6A**). Skin cells from a normal SCID mouse were propagated in plates for only a few passages after they were harvested as the sample of skin cells. Total RNAs were extracted from the skin cells, P0825, and M0825 cells, and cDNAs were synthesized. mRNA expression profiling between M0825 cells and P0825 cells, and with normal skin cells was compared (**Figure 6**). Comparison of growth factor-related genes EGFR and VEGFA, and oncogene KRAS indicated that their expression in M0825 was significantly lower than that in P0825 (**Figures 6B–E**). For the chemokine axis CXCL12-CXCR4 (**Figures 6F, G**), M0825 expressed significantly higher CXCL12, which also is a fibroblast marker. In **Figures 6H, I**, fibroblast marker ACTA2 (α-SMA) and Col1A1 did not show difference between M0825 and P0825, which confirmed that both subpopulations were mostly fibroblasts and fibroblast-like cells.

**Figure 7:**
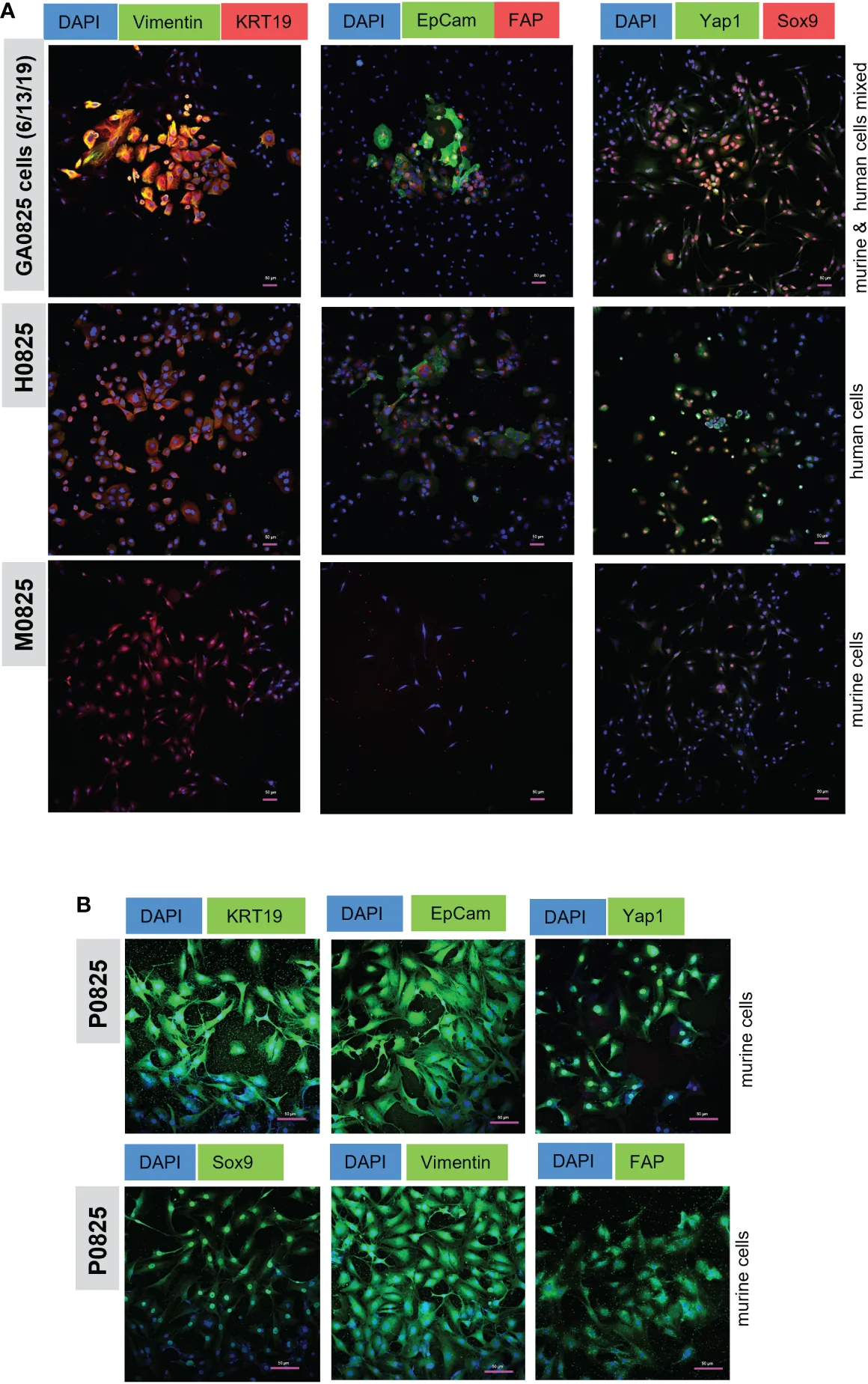
Immunofluorescence (IF) staining of cancer markers KRT19 (CK19) and EpCam, oncogene Yap1, stem cell marker Sox9, EMT marker Vimentin, and fibroblast marker FAP. **(A)** IF staining in human/mouse mixed pool GA0825 cells (6/13/2019) derived from GA0825-PDX-G1 (**Figure 4E**), human H0825 cells and murine M0825 cells. **(B)** mCherry labeled murine P0825 was stained with the same markers as in **(A)**, but second antibodies GFP^488^-conjugated Alex Fluor were used for green channel.

Compared to P0825, M0825 suffered haphazardly from selection bias that it did not contain cancer cells and/or cancer stem cells at all, but immortalized fibroblast-like cells. M0825 grew much slower than the main passaged murine P0825, indicating it lost its propagation potential (**Figure 2K**); moreover, M0825 did not carry cells expressing cancer and cancer stem markers as evidenced in **Figure 7A**.

### Murine P0825 and human H0825 retained cancer and stem cell markers

Human H0825 cells, murine P0825, and M0825 together with a GA0825-PDX-generated cell pool GA0825 cells (6/13/2019) were stained by IF for cancer markers, cancer stem cell markers, and fibroblast markers. GA0825 cells (6/13/2019) was subjected to intronic qPCR quantification and determined that it was a mixed pool of 84.3% murine and 15.7% human cells, which was used as a staining control (**Figure 4E**).

GA0825 cells, a mixture of human and murine cells, expressed high levels of cancer markers KRT19 and EpCam, and Sox9, a stem cell marker (**Figure 7A** top row). GA0825 also expressed medium levels of Vimentin and Yap1, a mesenchymal cell marker in epithelia to mesenchyme transition (EMT) and an oncogenic co-activator in the Hippo pathway, respectively. Compared to GA0825 cells, H0825 expressed moderately in KRT19, EpCam, Yap1, and Sox9 (**Figure 7A** middle row), though Yap1 expressed higher, which explained H0825 being weakly tumorigenic. Compared to GA0825 and H0825 cells, M0825 expressed negatively on all those markers (**Figure 7A** bottom row), since IF staining showed all the above markers worked for another murine P0825 cells (**Figure 7B**). M0825’s lack of cancer and stem cell markers was consistent to its non-tumorigenicity *in vivo*. The scale bar on the photos is 50 µm (**Figure 7A, B**).

In murine P0825 cells, due to its labeled mCherry-luciferase for the whole cell population and since the unlabeled was not available, a second antibody conjugated with GFP (Alexa Fluor 488) was used for detecting all the above markers, i.e., KRT19, EpCam, Yap1, Sox9, Vimentin and FAP (**Figure 7B**). P0825 expressed high levels of cancer markers KRT19 and EpCam, cancer stem marker Sox9, Hippo oncogenic co-activator Yap1, and EMT marker Vimentin. Positive FAP staining of P0825 indicated its fibroblast origin.

### WES analysis pointed to TP53 in GA0825-PDX-G1 as an important player for human-to-murine oncogenic transformation

**Figure 8:**
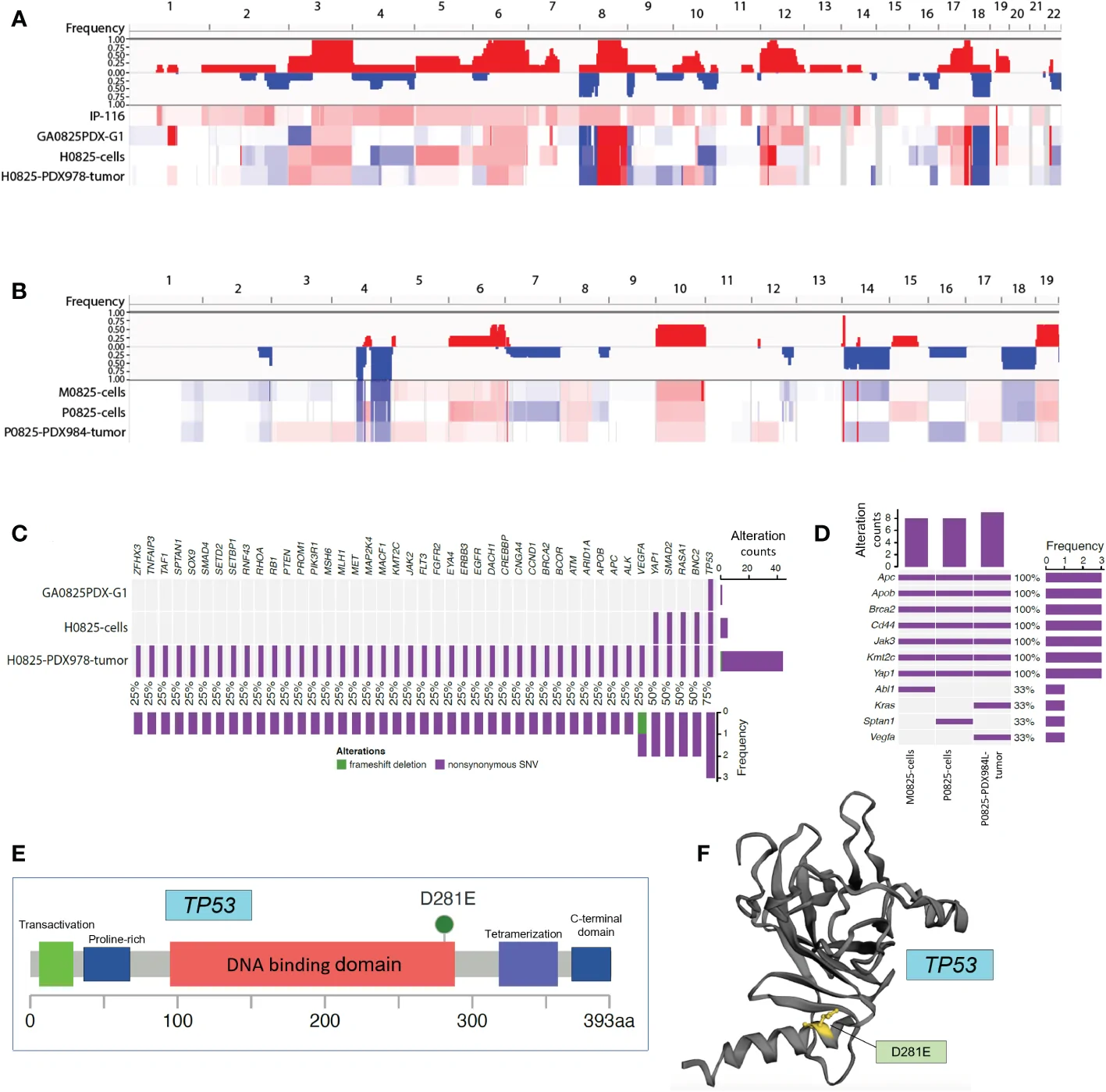
Whole exome sequencing (WES) analyses in patient ascites IP-116 and GA082517-PDX-derived human and murine samples. **(A)** Copy number variation (CNV) profiling for human lineage IP-116, GA0825PDX-G1 tumor, H0825 cells, and H0825-PDX978 tumor, with gains in red and losses in blue. **(B)** CNV profiling for murine lineage M0825 cells, P0825 cells, and P0825-PDX984L tumor. **(C)** Genomic mutations identified in human lineage GA0825PDX-G1, H0825 cells, and H0825-PDX978-tumor. The middle panel shows genomic mutations by sample and by gene (column). The histogram on the right shows the number of alterations accumulated on 43 listed genes in each individual sample. **(D)** Genomic alterations identified in murine lineage M0825 cells, P0825 cells, and P0825-PDX984 tumor. **(E)** Missense mutation in the TP53 from GA0825PDX-G1. **(F)** Mutated region mapped on the 3D structure of TP53.

There were two linages of samples for WES analysis. The human lineage went from the patient IP-116, GA0825-PDX-G1 tumor, H0825 cells to H0825-PDX978 tumor using patient IP-116 blood as the reference; and a second murine lineage went from the main passage P0825 cells, parallel murine subpopulation M0825 cells to P0825-PDX984 tumor using normal blood from a SCID mouse as the reference. Copy number variation (CNV) profiling of the whole genomes clearly showed that the patient ascites IP-116 was very different from the other three human samples (**Figure 8A**) in genome-wide CNV gains. The other three GA0825-PDX-G1 tumor, H0825 cells to H0825-PDX978 tumor showed similar patterns of genomic alterations, such as CNV gains in Chr3 and Chr6 and substantial losses and gains in Chr8 and Chr18 (**Figure 8A**). A rationale of explanation was that only a low percentage of cells in patient IP-116 ascites generated the GA0825-PDX-G1 tumor, and therefore the genomic landscape was substantially altered, which is partially reflected in substantial chromosomal aberrations in G-banded karyotyping of H0825 cells (**Figure 3D**) and very slow growth (**Figure 2K**). CNV profiling on the second murine lineage showed that the main passaged P0825, pooled small subpopulation M0825 and the aggressive P0825-PDX984 tumor shared a similar genomic pattern (**Figure 8B**), in which CNV losses in Chr1, 2, 4, 7, 14, 16 and 18 were observed as compared to G-banded karyotyping where Chr4 and 7 were monosomic (**Figure 3E**). CNV profiling observed gains in Chr6, 8, 10, 17, and 19. Compared to the other two samples, main passaged P0825 remained less in CNV losses and gains, which may have explained that P0825 grew faster than the human H0825 and murine M0825 (**Figure 2K**), and that P0825 was aggressively tumorigenic (**Figure 3A**).

When genomic mutations were analyzed, clinical sample patient IP-116 suffered bad reads in the whole genome, and it was excluded from analysis. For the whole exosome, the human ascites generated GA0825-PDX-G1 had just one TP53 mutation, which is considered one of the most important key tumor suppressors and the guardian of the genome. The TP53 mutation passed down from the PDX, to the PDX-generated H0825 cells and H0825-PDX978 tumor. One significant change was with passages moving on, the gene mutation list expanded to include TP53, BNC2, RASA1, Smad2, and Yap1 in H0825 cells, and a total of 43 genes mutated in H0825-PDX978 tumor (**Figure 8C**). This phenomenon suggested that TP53 mutation in the initial ascites-generated PDX may have caused a cascade of gene mutations in its derived cells or downstream PDX models. Among the altered genes, BNC2 is a putative tumor suppressor (31); RASA1 gene (Ras p21 protein activator 1) is the regulator of Ras oncogene, and mutation in RASA1 may turn on oncogenic pathways (32); Smad2 is a tumor suppressor; and Yap1 is an oncogenic co-activator in the Hippo pathway when overexpressed.

In the murine lineage, genomic mutations shared on three murine samples were APC, Apob, Brca2, CD44, Jak3, Kmt2c and Yap1, consistent to the notion that P0825 and M0825 were parallel subpopulations from a common passaged cell pool. P0825-PDX984L was the murine PDX generated by P0825 cells (**Figure 8D**). Interestingly, these mutated genes partially overlapped with but independent of the mutated genes of human H0825-PDX978 tumor. The murine aggressive P0825-PDX984L even introduced mutations of two genes KRAS and VEGFA, the former an important oncogene and the latter a growth factor, which further aggravated its tumorigenicity.

In the murine lineage, APC and Brca2 are tumor suppressors, Apob gene is related to Apobec1, an RNA editing enzyme used to fend off RNA retroviruses from evolutionary perspective; CD44 is a cancer stem cell marker; KMT2C’s function remains unclear but its mutation is frequently found in cancers; Jak3 is part of Jak/STAT axis and is involved in cytokine signaling of immunity; and Yap1 is an oncogenic co-activator in Hippo pathway.

As for TP53 in **Figure 8C**, D281E mutation lies within the DNA-binding domain of the protein (**Figures 8E, F**) (8) subcategorized as DNA contact class. D281E is a mutation from aspartic acid to glutamic acid, both of which are acidic amino acids, but a conspicuous difference is the mutation changes from a 4-carbon to a 5-carbon molecule, enlarging and bulging the α-helix DNA binding domain, and this structural change makes regulatory DNA contact difficult (**Figure 8F**). D281 is adjacent to R282, one of the eight most common amino acid alterations in TP53 mutations that make up 28% cancers for this gene. One shared common feature of TP53 mutations is that they fail to bind to DNA specifically at the TP53 DNA regulatory sequence (33). This mutation D281E was reported in some of the 10,225 patient samples from 32 cancers by analyzing The Cancer Genome Atlas (TCGA) database, but not as a dominant mutation (34). In breast cancer, somatic mutation D281E was detected and believed to cause TP53 functional loss (35).

Further analysis of WES mutation list (**Table 3**) revealed that in the human lineage, all GA082517PDX-G1, H0825 cells and H0825-978R tumor shared the TP53 D281E mutation, H0825 cells-generated PDX H0825-978R tumor gained an additional Q165K mutation, suggesting a TP53 progressive mutation. Comparing Yap1 mutation in human lineage (S340A in H0825 cells) to the murine lineage A204P in M0825 cells, P0825 cells, and P0825-generated PDX P0825-984L tumor, the mutations were sitting on different domains in Yap1, which obviously was not horizontally passed form human tumor to the murine stromal cells, but an independent mutation in the murine lineage (**Table 3**).

**Table 3:** Somatic mutation list of WES analyses in GA082517-PDX-G1-derived human H0825 cells, murine M0825, P0825 cells, and their direct PDX tumors corresponding to **Figures 6C**, **D**.

Human in Figure 8C								
Sample	chr	start	end	ref_allele	alt_allele	gene	Mutation	Amino acid
GA-082517PDX-G1	17	7577095	7577095	G	T	TP53	nonsyn. SNV	p.D281E
H0825-cells	17	7577095	7577095	G	T	TP53	nonsyn. SNV	p.D281E
H0825-978R-tumor	17	7577095	7577095	G	T	TP53	nonsyn. SNV	p.D281E
H0825-978R-tumor	17	7578437	7578437	G	T	TP53	nonsyn. SNV	p.Q165K
H0825-cells	11	102080281	102080281	T	G	YAP1	nonsyn. SNV	p.S340A
H0825-cells	18	45396895	45396895	T	C	SMAD2	nonsyn. SNV	p.I93V
H0825-cells	18	45396904	45396904	G	C	SMAD2	nonsyn. SNV	p.P90A
H0825-cells	5	86675600	86675600	A	G	RASA1	nonsyn. SNV	p.T846A
H0825-cells	5	86675613	86675613	A	G	RASA1	nonsyn. SNV	p.N850S
H0825-cells	9	16727866	16727866	T	C	BNC2	nonsyn. SNV	p.T101A
Mouse in Figure 8C								
sample	chr	start	end	ref_allele	alt_allele	gene	mutation	amino acid
P0825-cells	12	8001816	8001816	C	T	Apob	nonsyn. SNV	p.P1127L
M0825-cells	12	8001816	8001816	C	T	Apob	nonsyn. SNV	p.P1127L
P0825-cells	12	8015187	8015187	A	G	Apob	nonsyn. SNV	p.K4052R
P0825-984L-tumor	12	8001816	8001816	C	T	Apob	nonsyn. SNV	p.P1127L
P0825-cells	18	34315736	34315736	G	T	Apc	nonsyn. SNV	p.R1895L
M0825-cells	18	34315736	34315736	G	T	Apc	nonsyn. SNV	p.R1895L
P0825-984L-tumor	18	34315736	34315736	G	T	Apc	nonsyn. SNV	p.R1895L
P0825-cells	2	102831530	102831530	C	T	Cd44	nonsyn. SNV	p.S573N
M0825-cells	2	102831530	102831530	C	T	Cd44	nonsyn. SNV	p.S573N
P0825-984L-tumor	2	102831530	102831530	C	T	Cd44	nonsyn. SNV	p.S573N
P0825-984L-tumor	5	25287055	25287055	T	G	Kmt2c	nonsyn. SNV	p.N4251T
P0825-cells	5	25287055	25287055	T	G	Kmt2c	nonsyn. SNV	p.N4251T
M0825-cells	5	25287055	25287055	T	G	Kmt2c	nonsyn. SNV	p.N4251T
M0825-cells	5	25299705	25299705	A	G	Kmt2c	nonsyn. SNV	p.L3535P
P0825-cells	5	150550847	150550847	T	C	Brca2	nonsyn. SNV	p.L2405P
M0825-cells	5	150550847	150550847	T	C	Brca2	nonsyn. SNV	p.L2405P
P0825-984L-tumor	5	150550847	150550847	T	C	Brca2	nonsyn. SNV	p.L2405P
P0825-cells	8	71679870	71679870	T	G	Jak3	nonsyn. SNV	p.L293R
M0825-cells	8	71679870	71679870	T	G	Jak3	nonsyn. SNV	p.L293R
P0825-984L-tumor	8	71679870	71679870	T	G	Jak3	nonsyn. SNV	p.L293R
P0825-cells	8	71684276	71684276	G	T	Jak3	nonsyn. SNV	p.G699C
M0825-cells	8	71684276	71684276	G	T	Jak3	nonsyn. SNV	p.G699C
P0825-984L-tumor	8	71684276	71684276	G	T	Jak3	nonsyn. SNV	p.G699C
P0825-cells	9	7973829	7973829	C	G	Yap1	nonsyn. SNV	p.A204P
M0825-cells	9	7973829	7973829	C	G	Yap1	nonsyn. SNV	p.A204P

ref_allele, wild-type allele; alt_allele, altered allele; nonsyn. SNV, nonsynonymous single-nucleotide variation.

Since the genome in the untreated SCID mice was normal, in Trp53 (human TP53) and other genes mentioned above, a strong connection of murine stromal cells undergoing CNV aberrations and a cascade of other genes’ mutation being turned on would be driven by GA0825-PDX-G1’s oncogenic drivers. Being a guardian gene of the genome, the sole TP53 mutation in the whole genome of the ancestral GA0825-PDX-G1 may have played an important role in the cancer-stroma oncogenic transformation in murine stroma.

## Discussion

This study used the intronic genomic qPCR method to quantify the compositions of cell lines, PDXs, and PDX-derived cell populations. To our best knowledge, we are the first to use intronic genomic qPCR to quantify if a PDX tumor or a cell line is of human, murine, or a mixed composition, with high sensitivity and within a time frame of a few hours. Second, we traced the timeline of oncogenic transformation from a gastric cancer ascites IP-116 to PDX model GA0825-PDX, to a complete murine aggressively tumorigenic cell line P0825. Third, TP53 mutation was detected in human GA0825-PDX as a sole mutation in the whole human genome that may have played an important role for this human-to-mouse oncogenic transformation.

Substantial arguments over human-to-murine oncogenic transformation in this study are put forth: 1) if GA0825-PDX-G1-carried murine stromal cells such as fibroblast, CAF cells, MSC cells and immune cells would dominate over human ascites-derived cells and take over *in vitro* passages; however, those stromal cells would die off after only limited passages, let alone transform into an aggressively perpetual tumorigenic murine P0825 cell line; 2) if human GA0825-PDX-G1 cancer cells were contaminated in *in vitro* passages, but our lab was not growing any murine line for the whole period when the project of GA082516-PDX was being studied. The murine Kp-Luc2 and RAW264.7 lines were added much later for the purpose of developing the novel intronic qPCR method, yet Kp-Luc2 and RAW264.7 have completely different cell morphologies. An STR fingerprinting analysis of P0825 (06/23/21) proved that it is an uncontaminated murine line with its unique microsatellite patterns, unlike any of the ~400 commercial lines in the ATCC database; 3) if the newly developed intronic genomic qPCR in this study is not sensitive enough to pick up human Gapdh copies in the number of dozens or hundreds. Theoretically, qPCR could pick up a single DNA copy in optimum conditions. In this study, P0825 (06/23/21) quantified 99.9898% cells being murine and produced huge tumors in four SCID mice, while H0825 (0623/21) quantified 99.9945% cells being human yet produced very minimal tumors in four SCID mice. Tumors produced by P0825 in mice were all murine verified by intronic qPCR (**Figure 4B, C**). The compelling evidence concluded that murine P0825 (06/23/21) was oncogenically transformed by xenografted human ascites.

One would argue the TaqMan qPCR would provide better quantification for biosamples than the SYBR Green method, it is generally agreed that this would be the case. In our experiments, simpler SYBR Green qPCR worked well and is cheaper, since ΔCt difference of hGapdh/mGapdh detecting *bona fide* genomes *versus* nonspecific amplicons would be big enough to validate the composition of biosamples. TaqMan qPCR quantification is presented in **Supplementary Figure S4** to be compared to SYBR Green qPCR in **Figure 1F**. This TaqMan qPCR used the same sets of hGapdh and mGapdh primers as in the SYBR Green qPCRs, and two probes tested the sensitivity and quantification in human cell line AGS and murine line Kp-Luc2. The general patterns of linear increment and decrement of both methods were similar, and both detected 50 copies as sensitivity tests (circles). Yet, SYBR Green qPCR in **Figure 1F** performed better. In TaqMan qPCR, murine copies were overcounted and human copies were undercounted, a possible rationale would be that the added two probes with slight affinity difference when binding to the genomes in TaqMan skewed the results slightly. Therefore, the intronic genomic qPCRs throughout this study using SYBR Green remained a robust detection and quantification method.

One study in 2019 reported pediatric neuroblastoma PDXs using TaqMan relative mRNA expression to differentiate human or murine tumors (36), but this method would be problematic if the samples tested were mixed human/murine cells. Another recent report using TaqMan detecting DNAs of human and murine mitochondrial cytochrome c oxidase subunit I (MTCO1) gene was found to determine if human cancer cells were contaminated by murine cells with a sensitivity of 100 murine cells/1 million cancer cells (37). Yet, mitochondrial copies do not equate genomic copies; a human or murine cell has many more mitochondrial copies. We have not found whether there are other reports to quantify human or murine compositions of cell lines or PDX tumors in the PubMed website using simple yet accurate intronic genomic qPCR method.

This study has uncovered that host contamination is not a negligible phenomenon in the PDX models, as corroborated in lung cancer PDX-derived lines (10). Our institutional Cytogenetics and Cell Authentication Core (CCAC) stated that contamination rate of host cells in human PDX-derived cells was astoundingly high. Thus, detection of host contamination is imperative for cell lines, PDX models, PDX-derived cells for cancer research, and preclinical therapeutic studies.

In PDXs’ xenotransplanted with prostate tumor cell lines, human cancer cells transformed into oncogenic murine lines (5–7). There are other theories for the mechanisms: human-host cell fusion and horizontal signal transfer. *In vivo* fusion of the human tumor cells with hamster stromal cells and permanently transcribing human genes within have been reported using human glioblastoma-, lymphoma-hamster stromal cells. Karyotyping, PCRs and fluorescence *in situ* hybridization (FISH) analyses proved that spontaneous fusion of human tumor and host hamster cells *in vivo*, and certain human chromosomes and genes retained in the fused cells. Therefore, it was postulated that cell fusion caused the horizontal transmission of malignancy and donor genes to host stromal cells (38, 39). By karyology analysis, one of the hybrid tumors had a total of 15 human chromosomes in its cells. Cancer cells can transduce adjacent stromal cells, whose progeny permanently transcribed genes with malignant and other gene functions of the human donor DNAs. Using heterospecific *in vivo* cell fusion, genes encoding oncogenic and organogenic traits may be identified (40). Accumulating evidence suggested interactions between tumor cells and host cells in the TME became essential for tumor progression and metastases. Most evidence pointed to fusion phenomena, but others suggested that transfer of cell-free DNA (cfDNA) might play an important role. The evidence was: 1) the TME includes microvesicles, shed from tumor cells, which are important mediators of tumor growth, angiogenesis and metastasis; 2) gene transfer *via* the uptake of apoptotic bodies might also mediate transformation of normal host cells; 3) plasma from colon cancer patients transformed cultured NIH-3T3 cells, then generated carcinomas when injected into mice (41). In view of the above reports, this study has not detected genomic hGapdh and mGapdh copies simultaneously in pure human samples, pure murine samples, or transformed murine lines derived from PDXs, but this conclusion is solely based on detection and quantification using a single genomic Gapdh gene. However, our WES analysis may have pointed to the mutation of TP53 as a possible driver for human-to-mouse oncogenic transformation.

Possibly, human PDX engraftments such as tumors may exert similar cancer-stroma cell-to-cell crosstalk and pass genetic information onto stromal cells. As to how stromal cells were transformed by the PDX engraftment and turned on cancer-stroma oncogenic transformation, there is no report so far according to PubMed search, the mechanism remains unclear. In this study, TP53 mutation D281E was found as the sole mutation in the whole human genome of GA0825-PDX in a nude mouse, which not only was seen in its subsequent H0825 cells and H0825-PDX-978 tumor, but also convincingly turned on mutations in a cascade of other oncogenes and tumor suppressors in succession. It becomes credible that this tumor suppressor gene is the master gene and its mutation may have played a role in mutations of other genes. Moreover, given murine M0825, P0825 cells and P0825-PDX984L tumor had different set of genes with mutations, a rationale would be the stromal cells received oncogenic signal(s) at downstream of the cascade initiated by mutant TP53, but maybe not directly. Future research may reveal how TP53 mutation in human cancer cells in a PDX model passes down signals to murine stromal cells.

## Conclusions

We have developed a new authentication and quantification method termed as intronic genomic qPCR, whose primers situated on the 3^rd^ and the 4^th^ intron of the human Gapdh gene (hGapdh), and the 1^st^ intron and the 2^nd^ intron of the murine Gapdh gene (mGapdh) flanking respective exons. This intronic qPCR was able to quantify human/mouse genomic copies with high sensitivity and within a time frame of a few hours. Second, using this qPCR method, some commonly used gastric cancer lines and other lines were authenticated as being human or murine lines. Third, we have detected a human gastric cancer patient IP-116’s ascites-derived PDX model GA0825-PDX-G1 transformed and progressed from human/mouse mixture to a purely murine cell line P0825, which was verified by the sensitivity of this intronic genomic qPCR and STR fingerprinting analysis. This murine line P0825 was aggressively tumorigenic. mRNA expressions of P0825-PDXs in five mice were higher in tumors than the adjacent normal tissues in cancer markers, oncogenes, and tumor suppressors by all metrics. Fourth, immunofluorescence (IF) staining verified that in murine P0825 cells, cancer, and cancer stem markers were highly expressed compared to the other murine subpopulation M0825 and human H0825, which was consistent with those cells’ tumorigenic capabilities. Lastly, through WES analysis, it was discovered that in GA0825-PDX-G1, the TP53 was the solely mutated gene, which might have played a role in a cascade of genomic mutations related to cancer-stroma oncogenic transformation, which has shed new light towards tumorigenesis.

## Limitations of the study

The following limitations in this study need to be addressed: 1) This newly developed intronic qPCR method only authenticates and quantifies human and murine genomic copies based on the Gapdh gene, it does not detect genomic copies of other rodents or animals in biological and medical research, although the principle of designing species-specific intronic primers remains the same. 2) This intronic qPCR accurately detecting hGapdh and mGapdh genomic copies is on the basis that human and murine samples are in normal diploidy, i.e., one copy of genomic Gapdh per cell. Due to the heterogeneity of tumors, this method provides an estimated copy number detection. Moreover, in some highly genome-instable samples derived from malignant tumors, when chromosomal polyploidy, aneuploidy, monosomy, duplication, and deletion are frequent, this copy number will be inaccurate, despite the fact that it still answers unequivocally if the samples are human, murine or mixtures. 3) The clinical patient ascitic sample IP-116 suffered bad reads in WES capture, and thus was not included in the WES somatic mutation analysis due to its irreplaceable nature. Otherwise it would provide valuable information such as if its TP53 was mutated in D281E or not. 4) The complexity of neoplasia, oncogenesis, tumor metastasis, and their mechanisms have been a consensus knowledge in cancer research, a newly developed technique together with WES analysis revealing a conspicuous TP53 mutation in the human PDX model faced a difficult task of directly linking it to the murine stroma oncogenic transformation. Even TP53 mutations causing cancers has seen many publications in PubMed (42). 5) The human-murine oncogenic transformation could well be rare events when patient samples or patient-derived cells are injected into PDX models, although host cells carryover is a common phenomenon. It would be unrealistic to request this finding be duplicated in another PDX model in this study. As for the mechanism, more research is needed to prove if human tumors in PDX models could horizontally transfer oncogenic signals to host stromal cells, or human-host cell fusions are the causes.

## Data availability statement

The original contributions presented in the study are included in the article/**Supplementary Material**. Further inquiries can be directed to the corresponding authors.

## Ethics statement

All participants provided informed written consent, and the animal protocol and human subject protocol involved in this study was approved by the institutional Review Board of UT MD Anderson Cancer Center.

## Author contributions

JJ: Conceptualization, formal analysis, investigation, visualization, methodology, writing–original draft, writing–review and editing. LH: investigation, methodology, writing–review and editing. YF: investigation, methodology. RW: investigation, visualization, methodology. AS: resources. MP: resources. XY: resources. SSh: resources. LM: resources. MS: writing–review and editing, resources. KYa: resources. KYo: resources. BZ: resources. JW: resources. LW: supervision, methodology. SSo: supervision, fund acquisition, writing–review and editing, project administration. JA: supervision, fund acquisition, writing–review and editing, project administration. All authors contributed to the article and approved the submitted version.
