# Supplementary material for: A new intronic quantitative PCR method led to the discovery of transformation from human ascites to murine malignancy in a mouse model
Source: Front Oncol. 2023 Feb 6;13:1062424. doi: 10.3389/fonc.2023.1062424 (PMC9972586; doi:10.3389/fonc.2023.1062424)

Supporting information

Additional supporting information may be found online in the Supporting Information section at the end of the article.

Supplementary Table

Table S1. Samples and libraries for whole exosome sequencing (WES) analysis

Species	Samples for WES capture	Library
Human	Patient IP-116 blood	Agilent SureSelect Human All Exon V6
	Patient ascites IP-116	Agilent SureSelect Human All Exon V6
	GA0825-PDX-G1 tumor from IP-116 in a SCID mouse	Agilent SureSelect Human All Exon V6
	H0825 cells growing on plates	Agilent SureSelect Human All Exon V7
	H0825-PDX tumor (#978R) from SCID mice	Agilent SureSelect Human All Exon V7
Mouse	Normal blood from SCID mice	Agilent SureSelect Mouse All Exon V7
	P0825 cells growing on plates	Agilent SureSelect Mouse All Exon V7
	P0825 PDX tumor (#984L) from a SCID mouse	Agilent SureSelect Mouse All Exon V7
	M0825 cells growing on plates	Agilent SureSelect Mouse All Exon V7

Supplementary Figures

Fig. S1. Complete Genomic DNA sequence alignment of human hGapdh (NC_000012.12) versus mouse mGapdh (NC_000072.7)

```
#=======================================
# 1: hGpdh-NC_000012.12
# 2: mGapdh-NC_000072.7
# Identity:     2646/5314 (49.8%)
# Similarity:   2646/5314 (49.8%)
# Gaps:         2157/5314 (40.6%)
# Score: 8677.0
#=======================================

hGpdh-NC12.12      1 --------------------------------------------------      0

mGapdh-NC72.7      1 aacccttaagagggatgctgcccttaccccggggtcccagcttaggttca     50 Mouse Exon1

hGpdh-NC12.12      1 --------------------------------------------------      0

mGapdh-NC72.7     51 tcaggtaaactcaggagagtgtttgtaagtctcaattatcaggtttccag    100

hGpdh-NC12.12      1 --------------------------------------------------      0

mGapdh-NC72.7    101 gctgcagggcatcctgacctatggcgtagcaatctccttttcaagcgtct    150

hGpdh-NC12.12      1 --------------------------------------------------      0

mGapdh-NC72.7    151 tcctggccacccccttgccctcacagcgcggtacacaagccaggcctctg    200

hGpdh-NC12.12      1 --------------------------------------------------      0

mGapdh-NC72.7    201 gccaaagacagaagccaggaggggggggggggcagatagggaaatggggc    250

hGpdh-NC12.12      1 --------------------------------------------------      0

mGapdh-NC72.7    251 tcctgagggctcccgcgggaggaaaagtgcccccaccctggcattttctt    300

hGpdh-NC12.12      1 --------------------------------------------------      0

mGapdh-NC72.7    301 ccactcttttttttttcggggggggggttgctgtgtcactaccgaagaa    350

hGpdh-NC12.12      1 --------------------------------------------------      0
```

```
mGapdh-NC72.7     351 caacgaggagaagatcctcaacttttccgcagccttttcaataatgggga    400

hGpdh-NC12.12       1 --------------------------------------------------      0

mGapdh-NC72.7     401 gaggttcgatgatgcagtggcagggagacccacacttctccatttcccct    450

hGpdh-NC12.12       1 --------------------------------------------------      0

mGapdh-NC72.7     451 gttctcccattttactcgggaagcagcattcaggtctctgggtcctggat    500

hGpdh-NC12.12       1 --------------------------------------------------      0

mGapdh-NC72.7     501 gtccttggtgcacactccaaggactcctcgtccttaagttcatagtctgt    550

hGpdh-NC12.12       1 --------------------------------------------------      0

mGapdh-NC72.7     551 attccctgagtcctatcctgggaaccatcacccggtcacctcctgagcgg    600

hGpdh-NC12.12       1 --------------------------------------------------      0

mGapdh-NC72.7     601 ggcaatctcagctcccctcccccctatcagttcggagcccacacgcttggt    650

hGpdh-NC12.12       1 --------------------------------------------------      0

mGapdh-NC72.7     651 gcgtgcacatttcaaaaatgaggcgggtccaaagagagggaggaggggaa    700

hGpdh-NC12.12       1 --------------------------------------------------      0

mGapdh-NC72.7     701 atgagagaggcccagctactcgcggctttacgggtgcacgtagctcaggc    750

hGpdh-NC12.12       1 --------------------------------------------------      0

mGapdh-NC72.7     751 ctctgcgcccttgagctaggactggataagcagggcgggaggcggggcgc    800

hGpdh-NC12.12       1 --------------------------------------------------      0

mGapdh-NC72.7     801 gcgtcatcagctccccccaccatccgggttcctataaatacggactgca    850

hGpdh-NC12.12       1 ------------gctctctgctcct-cctgttcgacagtcagccgcatct     37
                                  ||||||||||||| |||||||.|.||.|.|||||||||
mGapdh-NC72.7     851 gccctccctggtgctctctgctcctccctgttccagagacggccgcatct    900

hGpdh-NC12.12      38 tcttttgc-gtcgccaggtgaaga-cgggcggagagaaacccgggaggct     85
                      ||||.||| || ||||||||||.| |  ||||||.|...|.|.||||||.
mGapdh-NC72.7     901 tcttgtgcagt-gccaggtgaaaatc--gcggagtgggccgcaggaggcc    947

hGpdh-NC12.12      86 agggacggcctgaa---ggcggcaggggcgggcgca-------ggcc---    122
                      .|||||.|.|.|||   |  ||.|||||.|.|.|||       ||.|
mGapdh-NC72.7     948 ggggacagtcggaaactg--ggaaggggagtgggcactgtacgggtctag    995

hGpdh-NC12.12     123 ggatgtgttcgcgccgctgcggggtgggc---ccgggc---ggcctccgc    166
                      ||||        ||.|.||||..|||.||   ||||.|   || ||||||
mGapdh-NC72.7     996 ggat--------gctggtgcgaagtgtgcaagccggacccagg-ctccgc   1036

hGpdh-NC12.12     167 attgcaggggcggg----cggaggacgtgatgcggcgc-gggctgggcat    211
                      ||||||||||.|||    .|||||||||||||.||||| .||| |||.||
mGapdh-NC72.7    1037 attgcaggggggggatgatggaggacgtgatggggcgcacggc-gggaat   1085

hGpdh-NC12.12     212 ggaggcctggtgggggaggggaggggaggcgtgtgtgtcggccggggcca    261
                      ||||||..||||||||||||||    ..||.|| |||||...| ||||||
mGapdh-NC72.7    1086 ggaggcggggtgggggagggga----ctgcctg-gtgtccttc-gggcca   1129
                                                                mGapdh.Int1.F→
hGpdh-NC12.12     262 ctaggc---gctcactgttc-tctccctccgcgcagccgagccacatcgc    307
                      |   ||   .|||| |.||| |||||     .||||||        |||
mGapdh-NC72.7    1130 c---gctaatctca-ttttcttctcc-----tgcagcc--------tcg-   1161

hGpdh-NC12.12     308 tc----agacaccatggggaaggtgaaggtcggagtcaacgggtgagttc    353
                      ||    |||||..||      ||||||||||||.||.|||||||||||||
mGapdh-NC72.7    1162 tcccgtagacaaaat------ggtgaaggtcggtgtgaacgggtgagttc   1205 Mouse Exon2

hGpdh-NC12.12     354 gcgggtggctggggggccctgggctgcgaccgcccccgaaccg-cgtcta    402
                      ..||      |.||||||||  |||               ||| .|.|||
mGapdh-NC72.7    1206 cagg------gcggggccct--gct---------------ccgttgccta   1232

hGpdh-NC12.12     403 cg--agccttgcgggctccgggtctttgcagtcgtatgggggcag-----    445
                      ||  .|.|||||.|.|.|.|||.||.||||||..|.|||||  ||
mGapdh-NC72.7    1233 cgcaggtcttgctgacccgggggctctgcagtactgtgggg--aggtgga   1280

hGpdh-NC12.12     446 ---ggtagctgttccccgcaaggagagctcaaggtcagcgctcggacctg    492
                         |||.||.|...|.|.||||||||.|||||||||||||||||||||||
mGapdh-NC72.7    1281 tgaggtggccgaagcgcccaaggagacctcaaggtcagcgctcggacctg   1330
                                                                 TaqMan mouse probe
hGpdh-NC12.12     493 gcggagccccgca---------cccaggctgtggcgcc-ctgtgcagctc    532
                      |||..|.|.||||         |||| ||||..||.|| ||| |.|.|||
mGapdh-NC72.7    1331 gcgatggctcgcacttgcggccccca-gctgctgcacctctg-gtaactc   1378
                                                                ← mGapdh.Int2.R
hGpdh-NC12.12     533 cgcccttgcggcgccat---ctgcccggagcctccttcccctagtcccca    579
                      ||||.||||||.|  ||   |.||||||||.||        ||      |
mGapdh-NC72.7    1379 cgcctttgcgggg--atgagcggcccggagtct--------ta------a   1412

hGpdh-NC12.12     580 gaaacaggaggtccctactcc---cgcccg---agatcccgacccgga--    621
                      |.|..||||    .|.||.||   ||||||   ||| |||..||||.|
mGapdh-NC72.7    1413 gtattagga----acaaccccacgcgcccgttcaga-cccatcccgtaat   1457

hGpdh-NC12.12     622 cccctaggtgggggacgctttctttcctttcgcgctctgcggggtcacgt    671
                      |||| ||...||||...||||||||.|||||||||.|||.||.|||||||
mGapdh-NC72.7    1458 cccc-agtccggggcttctttctttactttcgcgccctgaggagtcacgt   1506

hGpdh-NC12.12     672 gtc-gcagaggagcccctcccccacggcctccggcaccgcaggccc----    716
                      |.| |.||.|.|||||||||||||   .||||           |||
```

```
mGapdh-NC72.7   1507 gccaggagggaagcccctcccccca---tctcc-----------cccttcc   1542

hGpdh-NC12.12    717 -cgggat-gctagtgcgca--gcg------ggtgcatccctgtccggat-    755
                      ||||.| |   ||| |||  |||      |||||||.|||.|.||.||
mGapdh-NC72.7   1543 tcggggtcg---gtg-gcatggcgttgtgaggtgcatacctttgcgcatc   1588

hGpdh-NC12.12    756 ---------gctgcgcctgc------ggtaga---gcggccgccatgttg    787
                              |||..|.||.|      |||| |   ||.||||||||||||
mGapdh-NC72.7   1589 atctcccaggcttgggcttcctttagggta-actggccgccgccatgttg   1637

hGpdh-NC12.12    788 caaccgggaaggaaatgaatgggcagccgttaggaaa----------gcc    827
                     |||.|||||||||||||||||..|.|||||||.||||          |||
mGapdh-NC72.7   1638 caaacgggaaggaaatgaatgaaccgccgttatgaaatcttgcttaggcc   1687

hGpdh-NC12.12    828 t------------gccggtgactaaccctgcgctcctgcctc---gatgg    862
                     |            ||..|||||||||      |||.|.||||   |.|||
mGapdh-NC72.7   1688 ttccttcttcctagcttgtgactaac------ctcattcctctcggctgg   1731

hGpdh-NC12.12    863 gtggagtcgcgtgtggcggggaagtcaggtggagcgaggct---------    903
                     |||||||..|.|.|..|..|.|.|.||||||..||.|||||
mGapdh-NC72.7   1732 gtggagtgtcctttatcctgtaggccaggtgatgcaaggcttccgtgctc   1781

hGpdh-NC12.12    904 ---------------------agctg----------------------gc    910
                                          |.|||                      ||
mGapdh-NC72.7   1782 tcgagagagttctacctcacaatctgtctcaccttattagccttaaaagc   1831

hGpdh-NC12.12    911 cc--ga-----tttctcctccggg--tgatgcttttcctagattattctc    951
                     ||  ||     .||.|||| ||||  |.||||.|.|.|||||||||||||
mGapdh-NC72.7   1832 ccttgagccttattgtcct-cgggcataatgcgtattctagattattctc   1880

hGpdh-NC12.12    952 tggtaaatcaaagaagtgggtttatggaggtcctcttgtgtcccctcc--    999
                     | |.||||||   |||.||..|||..|||||||.||||    .|||||
mGapdh-NC72.7   1881 t-gaaaatca---aagcggacttacagaggtccgcttg----acctccca   1922

hGpdh-NC12.12   1000 -ccgcagaggt-gtggtggctgtggcatggtgccaagccgggaga-----   1042
                      ||.||||||| ||      |.||||.|.||||..|||||.|.||
mGapdh-NC72.7   1923 accccagaggtagt------tatggcgtagtgcagagccgtgggatgggg   1966

hGpdh-NC12.12   1043 agctgagtcatgggtagtt-ggaaaaggacatttccaccgcaaaatggcc   1091
                     ||||||||||| |||.||| .||||||.|..||||||||.|||||||||.
mGapdh-NC72.7   1967 agctgagtcat-ggtggttctgaaaagaaattttccaccacaaaatggct   2015

hGpdh-NC12.12   1092 cctctggtggtggccccttcctgcagcgccggctcacctcacggccccgc   1141
                     |||.|.||.|..|||||||||..|                    |||.||
mGapdh-NC72.7   2016 cctgtagtagcagccccttccatc--------------------ccctgc   2045

hGpdh-NC12.12   1142 ccttcccctgccagcctagcgttgaccc-gaccccaaaggccaggctgta   1190
                     .||||||.|..||||||.||..|||||| |.|||.|.||||||||.||||
mGapdh-NC72.7   2046 acttcccatcacagcctcgcactgacccaggccctataggccaggatgta   2095

hGpdh-NC12.12   1191 aatgtcaccgggaggattgggtgtctgggcgcctcggggaa-cctgccct   1239
                     ||.||||....||||||||||||||...|||||||  .||| ||||||||
mGapdh-NC72.7   2096 aaggtcattaagaggattgggtgtccctgcgcctc--agaatcctgccct   2143

hGpdh-NC12.12   1240 tctccccattccgtcttccggaaaccagatctc-ccaccgcaccctggtc   1288
                     |||||||.||||.||.|||.||||||||||||| ||||..|.|||||.||
mGapdh-NC72.7   2144 tctccccgttccatcctccagaaaccagatctctccactccgccctgatc   2193

hGpdh-NC12.12   1289 tgaggttaaatatagc--tgctgacctttctgtagctgggggcctgggct   1336
                     |||||||||||.||||  || |||||||||||.|.|| ||||.|||.||
mGapdh-NC72.7   2194 tgaggttaaatttagccgtg-tgacctttctggatct-ggggtctgagc-   2240

hGpdh-NC12.12   1337 ggggctctctcccatcccttctcccc--acacacatgcacttacctg-tg   1383
                      |||||||   ||| ||||.||||||  ||||||||        ||| ||
mGapdh-NC72.7   2241 -gggctct---cca-ccctgctccccctacacacat--------ctgttg   2277

hGpdh-NC12.12   1384 ctcccactcctgatttctg---gaaaagagctaggaaggacaggcaactt   1430
                     ||||..|| ||.||||.||   ||          ||||.|||||     |
mGapdh-NC72.7   2278 ctccggct-ctcatttttgcccga----------gaagaacagg-----t   2311

hGpdh-NC12.12   1431 g----gcaaatcaaagccctgggactagggggtt--aaaatacagcttcc   1474
                     |    ||.||  ..||||||||||.||  |||||  |||         ||
mGapdh-NC72.7   2312 gtttcgcgaa--cgagccctgggatta--gggttggaaa---------cc   2348

hGpdh-NC12.12   1475 cctcttcccacccg---ccccagtctctgtcccttttgtaggagggactt   1521
                     |     |||||..|   .|.|||  |||.||||.||.||..|||||||||
mGapdh-NC72.7   2349 c-----cccacatgttttctcag--tctttccccttagttcgagggactt   2391

hGpdh-NC12.12   1522 agag--aaggggtgggcttgccctgtccagttaatttctgacctttactc   1569
                     .|||  .|..|||||||..|||||||.|.|.|.| ..||||||||||..|
mGapdh-NC72.7   2392 ggaggacacaggtgggcccgccctgtgctgctca-cgctgacctttagcc   2440

hGpdh-NC12.12   1570 ctgccctttgagtttgatgatg-ctgagtgtacaagcgttttct-ccct-   1616
                     .|||||||||||.|||.||||| .||||||..|||.|     ||| ||||
mGapdh-NC72.7   2441 ttgccctttgagcttgctgatgaatgagttcacagg-----tctgccctg   2485

hGpdh-NC12.12   1617 ---aaagggtgcag-ctg------agctaggcagcagcaagcattcctgg   1656
                        |..|||||.|| |||      |||.|.||.|.|.|||.|.|.||.|
mGapdh-NC72.7   2486 tccagggggtgtagcctgaagtccagccatgctggaacaaacttcccag-   2534

hGpdh-NC12.12   1657 ggtggcat-agtg--gggtggtgaataccatgtacaaagcttgtgcccag   1703
                        ||||| ||||  ||| ||||      ||||.|.||||||||.|||||
mGapdh-NC72.7   2535 ---ggcatgagtgatggg-ggtg------atgtgccaagcttgtacccag   2574

hGpdh-NC12.12   1704 actgtgggtggcagtgccccacatggccgcttctcctggaag-ggcttcg   1752
                     ||||.||            ||.|..|||.|||||    .||| |.|||.|
mGapdh-NC72.7   2575 actgggg------------caaactgccacttct----taagagacttag   2608

hGpdh-NC12.12   1753 tatgactggggg------tgttgggc-agc-----cct------------   1778
                     .||||||.||.|      ||.|||| |||     |||
mGapdh-NC72.7   2609 aatgacttggaggaggtttgctgggcaagcaatcacctcttggacaggaa   2658
```

```
hGpdh-NC12.12   1779 -ggagccttca-gttgcagccatgcctt--aagccaggcca-gcct-ggc   1822
                      |.|.|||.|| .||..|.||.|||..|  |||||..|||| |||| |||
mGapdh-NC72.7   2659 agaaacctccactttataaccgtgctataaaagccctgccaggcctcggc   2708

hGpdh-NC12.12   1823 agggaagctcaagggagataaaattcaacctcttgggccctcctgggg--   1870
                          .||||||.|.|.||||||||..|.|||||.||.|.|.||.|||
mGapdh-NC72.7   2709 -----tgctcaaagaatataaaattagatctctttggactttctagggtg   2753

hGpdh-NC12.12   1871 ------gt------------------------------------------   1872
                           ||
mGapdh-NC72.7   2754 ggaacagtctatatttgggttgtacatccaagcattcaactagctttatt   2803

hGpdh-NC12.12   1873 -aaggagatgctg---------------------------cattcgccct   1894
                      ||||..||.|||                           ||||     |
mGapdh-NC72.7   2804 aaagggaattctgaacaaaacatgaacttcctgatgcatacatt-----t   2848

hGpdh-NC12.12   1895 cttaatg-----------------------------------gggaggtg   1909
                     | |||||                                   ||.|.||
mGapdh-NC72.7   2849 c-taatgtactgtgtctgcataaaggcttgtacctttgttgtggtacgt-   2896

hGpdh-NC12.12   1910 gcctag-----ggctg---------ctcacatat----------------   1929
                     ||.|||     |||||         |.|||.|||
mGapdh-NC72.7   2897 gcatagctgatggctgcaggttctccacacctatggtgcaacagtattcc   2946

hGpdh-NC12.12   1930 --tctggag---------gagcct---------------cccct------   1947
                       ||||.||         .|||||               |||.|
mGapdh-NC72.7   2947 actctgaagaacatgagatagcctggggctcactacagacccatgaggag   2996

hGpdh-NC12.12   1948 ------cctca--tgccttcttgcctcttgtctcttagatttggtcgtat   1989
                           .||||  |.||.|.||   ||||||||.|.||||||||.|||||
mGapdh-NC72.7   2997 ttctgatctcagctcccctgtt---tcttgtctttcagatttggccgtat   3043 Mouse Exon3

hGpdh-NC12.12   1990 tgggcgcctggtcaccagggctgcttttaactctggtaaagtggatattg   2039
                     ||||||||||||||||||||||||..||..|..|||.||||||||.||||
mGapdh-NC72.7   3044 tgggcgcctggtcaccagggctgccatttgcagtggcaaagtggagattg   3093

hGpdh-NC12.12   2040 ttgccatcaatgaccccttcattgacctcaactacatggtgagtgctaca   2089 Start human ATG
                     ||||||||||.||||||||||||||||||||||||||
mGapdh-NC72.7   3094 ttgccatcaacgaccccttcattgacctcaactacatg-------------   3130

hGpdh-NC12.12   2090 tggtgagccccaaagctggtgtgggaggagccacctggctgatgggcagc   2139

mGapdh-NC72.7   3131 --------------------------------------------------   3130

hGpdh-NC12.12   2140 cccttcataccctcacgtattcccccaggtttacatgttccaatatgatt   2189 Human Exon2
                                                 ||.|||||||||||.|||||.|
mGapdh-NC72.7   3131 ----------------------------gtctacatgttccagtatgact   3153 Mouse Exon3

hGpdh-NC12.12   2190 ccacccatggcaaattccatggcaccgtcaaggctgagaacgggaagctt   2239
                     ||||.||.|||||||||.|.|||||.||||||||.|||||.|||||||||
mGapdh-NC72.7   3154 ccactcacggcaaattcaacggcacagtcaaggccgagaatgggaagctt   3203

hGpdh-NC12.12   2240 gtcatcaatggaaatcccatcaccatcttccaggagtgagtggaagacag   2289
                     ||||||||.||.||.||||||||||||||||||||
mGapdh-NC72.7   3204 gtcatcaacgggaagcccatcaccatcttccagga---------------   3238

hGpdh-NC12.12   2290 aatggaagaaatgtgctttggggaggcaactaggatggtgtggctccctt   2339

mGapdh-NC72.7   3239 --------------------------------------------------   3238

hGpdh-NC12.12   2340 gggtatatggtaaccttgtgtccctcaatatggtcctgtccccatctccc   2389

mGapdh-NC72.7   3239 --------------------------------------------------   3238

hGpdh-NC12.12   2390 ccccacccccataggcgagatccctccaaaatcaagtggggcgatgctgg   2439 Human Exon3
                                   ||||||.|||.|.||.|||||.|||||.||.||.||
mGapdh-NC72.7   3239 --------------gcgagaccccactaacatcaaatggggtgaggccgg   3274 Mouse Exon3

hGpdh-NC12.12   2440 cgctgagtacgtcgtggagtccactggcgtcttcaccaccatggagaagg   2489
                     .||||||||.|||||||||||.|||||.||||||||||||||||||||||
mGapdh-NC72.7   3275 tgctgagtatgtcgtggagtctactggtgtcttcaccaccatggagaagg   3324

hGpdh-NC12.12   2490 ctggggtgagtgcaggagggcccgcggg--aggggaagctgac--tcagc   2535
                     |.|||||.||||           ||.||  ||..||||.||||  .||.|
mGapdh-NC72.7   3325 ccggggtaagtg-----------gccggaaagctgaaggtgacgggcacc   3363
                     hGapdh.int3.F→
hGpdh-NC12.12   2536 cctgcaaag--gcaggacccgggttc--ataact--gtctg-------ct   2572
                     |.||..|.|  |||  |||.|....|  |.||||  |||||       ||
mGapdh-NC72.7   3364 cttgatatggtgca--acctgaaaaccaagaactgagtctgaaatcaact   3411 TaqMan human probe

hGpdh-NC12.12   2573 tctctgctgt----aggctcatttgcaggggggagccaaaagggtcatca   2618 human Exon4
                     |||.|.|..|    ||||.||.|||.||||.|||||||||||||||||||
mGapdh-NC72.7   3412 tctttcccttaaacaggcccacttgaagggtggagccaaaagggtcatca   3461 Mouse Exon4

hGpdh-NC12.12   2619 tctctgccccctctgctgatgcccccatgttcgtcatgggtgtgaaccat   2668
                     ||||.|||||.|||||.||||||||||||||.||.||||||||||||||.
mGapdh-NC72.7   3462 tctccgccccttctgccgatgcccccatgtttgtgatgggtgtgaaccac   3511
                                                   ←hGapdh.it4.R
hGpdh-NC12.12   2669 gagaagtatgacaacagcctcaagatcatcaggtgaggaaggcagggccc   2718
                     |||||.|||||||||...|||||||
mGapdh-NC72.7   3512 gagaaatatgacaactcactcaaga-------------------------   3536

hGpdh-NC12.12   2719 gtggagaagcggccagcctggcaccctatggacacgctcccctgacttgc   2768

mGapdh-NC72.7   3537 --------------------------------------------------   3536

hGpdh-NC12.12   2769 gccccgctccctctttctttg-cagcaatgcctcctgcaccaccaactgc   2817 human Exon5
                                       ||| |||||||||.||||||||||||||||||
mGapdh-NC72.7   3537 ------------------ttgtcagcaatgcatcctgcaccaccaactgc   3568
```

```
hGpdh-NC12.12  2818 ttagcacccctggccaaggtcatccatgacaactttggtatcgtggaagg  2867
                    |||||.||||||||||||||||||||||||||||||||.||.||||||||
mGapdh-NC72.7  3569 ttagccccccctggccaaggtcatccatgacaactttggcattgtggaagg  3618 Mouse Exon4

hGpdh-NC12.12  2868 actcatggtatgagagctggggaatgggactgag---gctcccacctttc  2914
                    .|||||||||||     |.||.|.||||   |||   ||||...|.||||
mGapdh-NC72.7  3619 gctcatggtatg-----taggcagtggg---gagacagctcatgcatttc  3660

hGpdh-NC12.12  2915 tcatccaagactggctcctccctgccggggctgcgtgcaaccctggggtt  2964
                    |.|||..|           |||||||    .||.|||.|.||
mGapdh-NC72.7  3661 ttatctta-----------ccctgcc----atgagtggaccc--------  3687

hGpdh-NC12.12  2965 gggggttctggggactggctttcccataatttcctttcaaggtggggagg  3014
                         ||||..|   |.|.|.||||    |||          ||
mGapdh-NC72.7  3688 -----ttctttg---taggtgtccc----ttt----------tg------  3709

hGpdh-NC12.12  3015 gaggtagaggggtgatgtggggagtacgctgcagggcctcactc------  3058
                      ||||||||||||            |..||||||.||||||||
mGapdh-NC72.7  3710 --ggtagaggggtg------------ccgtgcaggacctcactcattgcc  3745

hGpdh-NC12.12  3059 -----cttttgcagaccacagtccatgccatcactgccacccagaagact  3103 Human Exon6
                         .||||..||||||||||||||||||||||||||||||||||||||
mGapdh-NC72.7  3746 cccgtgttttctagaccacagtccatgccatcactgccacccagaagact  3795 Mouse Exon5

hGpdh-NC12.12  3104 gtggatggcccctccgggaaactgtggcgtgatggccgcggggctctcca  3153
                    ||||||||||||||.||.||.|||||||||||||||||.||||||..|||
mGapdh-NC72.7  3796 gtggatggcccctctggaaagctgtggcgtgatggccgtggggctgccca  3845

hGpdh-NC12.12  3154 gaacatcatccctgcctctactggcgctgccaaggctgtgggcaaggtca  3203
                    |||||||||||||||.||.|||||.|||||||||||||||||||||||||
mGapdh-NC72.7  3846 gaacatcatccctgcatccactggtgctgccaaggctgtgggcaaggtca  3895

hGpdh-NC12.12  3204 tccctgagctgaacgggaagctcactggcatggccttccgtgtccccact  3253
                    ||||.||||||||||||||||||||||||||||||||||||||.||.||.
mGapdh-NC72.7  3896 tcccagagctgaacgggaagctcactggcatggccttccgtgttcctacc  3945

hGpdh-NC12.12  3254 gccaacgtgtcagtggtggacctgacctgccgtctagaaaaacct-----  3298
                    .||||.|||||.||.|||||.|||||.|||||.||.||.||||||
mGapdh-NC72.7  3946 cccaatgtgtccgtcgtggatctgacgtgccgcctggagaaacctgtatg  3995

hGpdh-NC12.12  3299 --------------------------------------------------  3298

mGapdh-NC72.7  3996 tatggggagagctgggcttgtctctggtgtgacagtgacttgggacaagg  4045

hGpdh-NC12.12  3299 -----------------------------gccaaatatgatgacatcaag  3319 Human Ex6
                                                 |||||.|||||||||||||||
mGapdh-NC72.7  4046 atagtcattttggggtttgttcttatcaggccaagtatgatgacatcaag  4095 Mouse Exon6

hGpdh-NC12.12  3320 aaggtggtgaagcaggcgtcggagggccccctcaagggcatcctgggcta  3369
                    |||||||||||||||||.||.||||||||.||.|||||||||.|||||||
mGapdh-NC72.7  4096 aaggtggtgaagcaggcatctgagggcccactgaagggcatcttgggcta  4145

hGpdh-NC12.12  3370 cactgagcaccaggtggtctcctctgacttcaacagcgacacccactcct  3419
                    |||||||.|||||||.|||||||..||||||||||||.||.|||||||.|
mGapdh-NC72.7  4146 cactgaggaccaggttgtctcctgcgacttcaacagcaactcccactctt  4195

hGpdh-NC12.12  3420 ccacctttgacgctggggctggcattgccctcaacgaccactttgtcaag  3469
                    |||||||.||.||.||||||||||||||.|||||.|||.|||||||||||
mGapdh-NC72.7  4196 ccaccttcgatgccggggctggcattgctctcaatgacaactttgtcaag  4245

hGpdh-NC12.12  3470 ctcatttcctggtatgt--ggctggggccagaga-ctggctcttaaaaag  3516
                    |||||||||||||||||  ||.|||     ||.| |||.||.||   |||
mGapdh-NC72.7  4246 ctcatttcctggtatgtggggatgg-----gaaacctgacttttt---aag  4287

hGpdh-NC12.12  3517 tgca-----gggtctggcgccctctggtggctggctcagaaaaagggccc  3561
                    .|||     ||||.|||.||||||||||||||.||||||||||....|||
mGapdh-NC72.7  4288 agcaactgggggtttggtgccctctggtggctagctcagaaaagaaaccc  4337

hGpdh-NC12.12  3562 tgac-aactcttttc-----atcttctaggtatgacaacgaatttggcta  3605 human Exon7
                    ..|| |||..||.||     .|.||||||||||||||||.||||..|||||
mGapdh-NC72.7  4338 aaactaacagttgtcccaatttgttctaggtatgacaatgaatacggcta  4387 Mouse Exon7

hGpdh-NC12.12  3606 cagcaacagggtggtggacctcatggcccacatggcctccaaggagtaag  3655
                    ||||||||||||||||||||||||||||.|||||||||||||||||||||
mGapdh-NC72.7  4388 cagcaacagggtggtggacctcatggcctacatggcctccaaggagtaag  4437

hGpdh-NC12.12  3656 -acccctggaccaccagccccagcaagagcacaagaggaagagagagacc  3704
                     |.||||||||||||..||||||||||..||| .|||.|  ||||||.||
mGapdh-NC72.7  4438 aaaccctggaccacccaccccagcaaggacac-tgagca--agagaggcc  4484

hGpdh-NC12.12  3705 ctcactgctggggagtccctgccacactcagtcccccaccacactga--a  3752
                    ||            .||||     .|||| |.||||||  |||||||  |
mGapdh-NC72.7  4485 ct------------atccc-----aactc-ggccccca--acactgagca  4514

hGpdh-NC12.12  3753 tctcccctcctcacagttgccat-gtagacccc-----ttgaagaggga  3796
                    ||||    |||||||.||.|||| ..|||||||     |  ||.|  |||
mGapdh-NC72.7  4515 tctc----cctcacaatttccatcccagacccccataat--aaca--gga  4556

hGpdh-NC12.12  3797 ggggcctagggagccgcacct--tgtcatg--taccatcaataaagtacc  3842
                    |||||||||||||||...|||  |.||.||  |||||||||||||||.|.
mGapdh-NC72.7  4557 ggggcctagggagccctccctactctcttgaataccatcaataaagttcg  4606

hGpdh-NC12.12  3843 ctgtgctcaacca-  3855
                    |||    ||.|||
mGapdh-NC72.7  4607 ctg----cacccac  4616
```

Fig. S2. Genomic DNA sequence alignment of hGapdh with mGapdh around mouse intronic qPCR primer sets and human intronic qPCR primer sets. A. Alignment showing murine mGapdh primers and the TaqMan probe; B. Alignment showing human hGapdh primers and the TaqMan probe;

```
hGpdh-NC12.12    167 attgcaggggcggg----cggaggacgtgatgcggcgc-gggctgggcat    211
                     ||||||||||.|||    .|||||||||||||.||||| .||| |||.||
mGapdh-NC72.7   1037 attgcaggggggggatgatggaggacgtgatggggcgcacggc-gggaat   1085

hGpdh-NC12.12    212 ggaggcctggtggggagggggaggggaggcgtgtgtgtcggccggggcca    261
                     ||||||..||||||||||||||    ..||.|| |||||...| ||||||
mGapdh-NC72.7   1086 ggaggcggggtggggaggggga----ctgcctg-gtgtccttc-gggcca   1129
                                                        mGapdh.Int1.F→
hGpdh-NC12.12    262 ctaggc---gctcactgttc-tctccctccgcgcagccgagccacatcgc    307
                     |   ||   .|||| |.||| |||||     .||||||        ||| 
mGapdh-NC72.7   1130 c---gctaatctca-ttttcttctcc-----tgcagcc--------tcg-   1161

hGpdh-NC12.12    308 tc----agacaccatggggaaggtgaaggtcggagtcaacgggtgagttc    353
                     ||    |||||..||      ||||||||||||.||.|||||||||||||
mGapdh-NC72.7   1162 tcccgtagacaaaat------ggtgaaggtcggtgtgaacgggtgagttc   1205 Mouse Exon2

hGpdh-NC12.12    354 gcgggtggctggggggccctgggctgcgaccgcccccgaaccg-cgtcta    402
                     ..||      |.||||||||  |||               ||| .|.|||
mGapdh-NC72.7   1206 cagg------gcggggccct--gct---------------ccgttgccta   1232

hGpdh-NC12.12    403 cg--agccttgcgggctccgggtctttgcagtcgtatgggggcag-----    445
                     ||  .|.|||||.|.|.|.|||.||.||||||..|.|||||  ||     
mGapdh-NC72.7   1233 cgcaggtcttgctgacccgggggctctgcagtactgtgggg--aggtgga   1280

hGpdh-NC12.12    446 ---ggtagctgttccccgcaaggagagctcaaggtcagcgctcggacctg    492
                        |||.||.|...|.|.||||||||.|||||||||||||||||||||||
mGapdh-NC72.7   1281 tgaggtggccgaagcgcccaaggagacctcaaggtcagcgctcggacctg   1330
                                                       TaqMan mouse probe
hGpdh-NC12.12    493 gcggagccccgca---------cccaggctgtggcgcc-ctgtgcagctc    532
                     |||..|.|.||||         |||| ||||..||.|| ||| |.|.|||
mGapdh-NC72.7   1331 gcgatggctcgcacttgcggccccca-gctgctgcacctctg-gtaactc   1378
                                                         ← mGapdh.Int2.R
hGpdh-NC12.12    533 cgcccttgcggcgccat---ctgcccggagcctccttcccctagtcccca    579
                     ||||.||||||.|  ||   |.||||||||.||        ||      |
mGapdh-NC72.7   1379 cgcctttgcgggg--atgagcggcccggagtct--------ta------a   1412

hGpdh-NC12.12    580 gaaacaggaggtccctactcc---cgcccg---agatcccgacccgga--    621
                     |.|..||||    .|.||.||   ||||||   ||| |||..||||.|  
mGapdh-NC72.7   1413 gtattagga----acaaccccacgcgcccgttcaga-cccatcccgtaat   1457
```

A. Alignment of human hGapdh (NC_000012.12) and mouse mGapdh (NC_000072.7), yellow highlighted are the mouse intronic qPCR primers, gray highlighted is the TaqMan mouse probe. Blue highlighted is the mouse Exon2, human Exon2 is out of this alignment.

```
mGapdh-NC72.7   3239 --------------------------------------------------   3238

hGpdh-NC12.12   2390 ccccacccccatagcgagatccctccaaaatcaagtggggcgatgctgg   2439 Human Exon3
                                   ||||||.|||.|.||.|||||.|||||.||.||.||
mGapdh-NC72.7   3239 --------------gcgagaccccactaacatcaaatggggtgaggccgg   3274 Mouse Exon3

hGpdh-NC12.12   2440 cgctgagtacgtcgtggagtccactggcgtcttcaccaccatggagaagg   2489
                     .||||||||.|||||||||||.|||||.||||||||||||||||||||||
mGapdh-NC72.7   3275 tgctgagtatgtcgtggagtctactggtgtcttcaccaccatggagaagg   3324

hGpdh-NC12.12   2490 ctggggtgagtgcaggagggcccgcggg--aggggaagctgac--tcagc   2535
                     |.|||||.||||           ||.||  ||..||||.||||  .||.|
mGapdh-NC72.7   3325 ccggggtaagtg-----------gccggaaagctgaaggtgacgggcacc   3363
                     hGapdh.int3.F→
hGpdh-NC12.12   2536 cctgcaaag--gcaggacccgggttc--ataact--gtctg-------ct   2572
                     |.||..|.|  |||  |||.|....|  |.||||  |||||       ||
mGapdh-NC72.7   3364 cttgatatggtgca--acctgaaaaccaagaactgagtctgaaatcaact   3411 TaqMan human probe

hGpdh-NC12.12   2573 tctctgctgt----aggctcatttgcaggggggagccaaaagggtcatca   2618 human Exon4
                     |||.|.|..|    ||||.||.|||.||||.|||||||||||||||||||
mGapdh-NC72.7   3412 tctttcccttaaacaggcccacttgaaggtggagccaaaagggtcatca   3461 Mouse Exon4

hGpdh-NC12.12   2619 tctctgccccctctgctgatgcccccatgttcgtcatgggtgtgaaccat   2668
                     ||||.|||||.|||||.||||||||||||||.||.||||||||||||||.
mGapdh-NC72.7   3462 tctccgccccttctgccgatgcccccatgtttgtgatgggtgtgaaccac   3511
                                                          ←hGapdh.it4.R
hGpdh-NC12.12   2669 gagaagtatgacaacagcctcaagatcatcaggtgaggaaggcagggccc   2718
                     |||||.|||||||||...|||||||
mGapdh-NC72.7   3512 gagaaatatgacaactcactcaaga-------------------------   3536

hGpdh-NC12.12   2719 gtggagaagcggccagcctggcaccctatggacacgctcccctgacttgc   2768

mGapdh-NC72.7   3537 --------------------------------------------------   3536
```

B. Alignment of human hGapdh (NC_000012.12) and mouse mGapdh (NC_000072.7), yellow highlighted are the human intronic qPCR primers, gray highlighted is the TaqMan human probe. Green and blue highlighted are the human and the mouse Exon4, respectively.

Fig. S3. Protein sequence alignment of hGapdh (NP_002037.2) versus mGapdh (NP_001276655)

```
#=======================================
# Aligned_sequences: 2
# 1: hGapdh NP_002037.2
# 2: mGapdh NP_001276655
# Matrix: EBLOSUM62
# Gap_penalty: 10.0
# Extend_penalty: 0.5
# Length: 359
# Identity:     314/359 (87.5%)
# Similarity:   326/359 (90.8%)
# Gaps:          24/359 ( 6.7%)
# Score: 1619.0
#=======================================
hGapdh-NP2037.2       1 -----------------------MGK-VKVGVNGFGRIGRLVTRAAFNSG     26
                                               :.| |||||||||||||||||||..||
mGapdh-NP1276655      1 MLPLPRGPSLGSSGKLRRVFPRPVDKMVKVGVNGFGRIGRLVTRAAICSG     50

hGapdh-NP2037.2      27 KVDIVAINDPFIDLNYMVYMFQYDSTHGKFHGTVKAENGKLVINGNPITI     76
                        ||:|||||||||||||||||||||||||||:||||||||||||||.||||
mGapdh-NP1276655     51 KVEIVAINDPFIDLNYMVYMFQYDSTHGKFNGTVKAENGKLVINGKPITI    100

hGapdh-NP2037.2      77 FQERDPSKIKWGDAGAEYVVESTGVFTTMEKAGAHLQGGAKRVIISAPSA    126
                        ||||||:.||||:|||||||||||||||||||||||:|||||||||||||
mGapdh-NP1276655    101 FQERDPTNIKWGEAGAEYVVESTGVFTTMEKAGAHLKGGAKRVIISAPSA    150

hGapdh-NP2037.2     127 DAPMFVMGVNHEKYDNSLKIISNASCTTNCLAPLAKVIHDNFGIVEGLMT    176
                        ||||||||||||||||||||:|||||||||||||||||||||||||||||
mGapdh-NP1276655    151 DAPMFVMGVNHEKYDNSLKIVSNASCTTNCLAPLAKVIHDNFGIVEGLMT    200
```

```
hGapdh-NP2037.2     177 TVHAITATQKTVDGPSGKLWRDGRGALQNIIPASTGAAKAVGKVIPELNG    226
                        ||||||||||||||||||||||||||.|||||||||||||||||||||||
mGapdh-NP1276655    201 TVHAITATQKTVDGPSGKLWRDGRGAAQNIIPASTGAAKAVGKVIPELNG    250

hGapdh-NP2037.2     227 KLTGMAFRVPTANVSVVDLTCRLEKPAKYDDIKKVVKQASEGPLKGILGY    276
                        |||||||||||.||||||||||||||||||||||||||||||||||||||
mGapdh-NP1276655    251 KLTGMAFRVPTPNVSVVDLTCRLEKPAKYDDIKKVVKQASEGPLKGILGY    300

hGapdh-NP2037.2     277 TEHQVVSSDFNSDTHSSTFDAGAGIALNDHFVKLISWYDNEFGYSNRVVD    326
                        ||.||||.||||::|||||||||||||||:|||||||||||:||||||||
mGapdh-NP1276655    301 TEDQVVSCDFNSNSHSSTFDAGAGIALNDNFVKLISWYDNEYGYSNRVVD    350

hGapdh-NP2037.2     327 LMAHMASKE    335
                        |||:|||||
mGapdh-NP1276655    351 LMAYMASKE    359
```

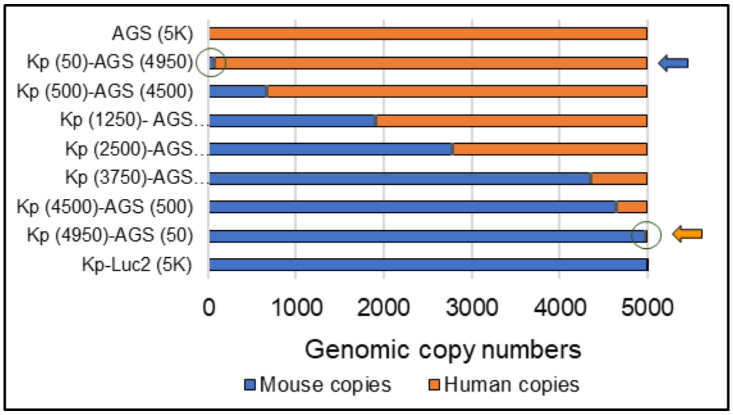

Fig. S4. TaqMan qPCR quantification as compared to SYBR Green qPCR in Fig. 1F. TaqMan qPCR used the same sets of hGapdh, mGapdh primers as the SYBR Green qPCR, and two probes tested the sensitivity and quantification in human cell line AGS and murine line Kp-Luc2. The copy number mixtures were the same as in Fig. 1F. Primers and probes are listed in Table 1. Arrows point to the circles where 50 copies of human genome and murine genome were visualized.

Suppl. Doc 1

08/09/2021 SYBR qPCR for P0825, M0825 and H0825

Well	Well Positi	Sample Na	Target Nar	CT
1	A1	P0825	mGAPDH	19.352
2	A2	M0825	mGAPDH	18.763
3	A3	H0825	mGAPDH	32.254
13	B1	P0825	mGAPDH	19.490
14	B2	M0825	mGAPDH	18.775
15	B3	H0825	mGAPDH	34.502
25	C1	P0825	mGAPDH	19.312
26	C2	M0825	mGAPDH	18.790
27	C3	H0825	mGAPDH	34.435
37	D1	P0825	hGAPDH	33.037
38	D2	M0825	hGAPDH	23.957
39	D3	H0825	hGAPDH	19.794
49	E1	P0825	hGAPDH	32.590
50	E2	M0825	hGAPDH	23.880
51	E3	H0825	hGAPDH	19.501
61	F1	P0825	hGAPDH	32.315
62	F2	M0825	hGAPDH	23.792
63	F3	H0825	hGAPDH	19.454

1. Raw qPCR data ↑

	P0825	M0825	H0825
mGAPDH	19.352	18.763	32.254
mGAPDH	19.490	18.775	34.502
mGAPDH	19.312	18.790	34.435
hGAPDH	33.037	23.957	19.794
hGAPDH	32.590	23.880	19.501
hGAPDH	32.315	23.792	19.454

2. Layout ↑

3. Calculate →

	8/9/2021		
	P0825	M0825	H0825
hGapdh	33.037	23.957	19.794
hGapdh	32.590	23.880	19.501
hGapdh	32.315	23.792	19.454
mGapdh	19.352	18.763	32.254
mGapdh	19.490	18.775	34.502
mGapdh	19.312	18.790	34.435
hGapdh	33.037	23.957	19.794
hGapdh	32.590	23.880	19.501
hGapdh	32.315	23.792	19.454
mGapdh	19.352	18.763	32.254
mGapdh	19.490	18.775	34.502
mGapdh	19.312	18.790	34.435
hGapdh	32.648	23.876	19.5830307
mGapdh	19.384	18.776	33.73031235
ΔCt h-m	13.653	5.181	-13.936
ΔCt h-m	13.206	5.104	-14.229
ΔCt h-m	12.931	5.016	-14.276
	P0825	M0825	H0825
ΔCt m-h	-13.296	-5.114	12.671
ΔCt m-h	-13.1579876	-5.10111	14.91867065
ΔCt m-h	-13.335577	-5.0866	14.85210037
	P0825	M0825	H0825
power h-m	7.76276E-05	0.02756	15676.84868
power h-m	0.000105856	0.029082	19202.8459
power h-m	0.000128065	0.030899	19844.67703
	P0825	M0825	H0825
Power m-h	10057.24842	34.62079	0.00015333
Power m-h	9140.057935	34.32314	3.22874E-05
Power m-h	10337.33736	33.97964	3.38121E-05
	P0825	M0825	H0825
h-m average	0.00010385	0.029181	18241.4572
m-h average	9844.881237	34.30786	7.31431E-05
	P0825	M0825	H0825
h-Percentage	0.007762156	2.682127	99.99362157
h-Percentage	0.010584468	2.826004	99.99479271
h-Percentage	0.012804909	2.997309	99.99496112
	P0825	M0825	H0825
m-Percentage	99.99005791	97.19265	0.015330633
m-Percentage	99.98906035	97.16899	0.003228633
m-Percentage	99.99032726	97.14119	0.003381097
	P0825	M0825	H0825
hGapdh	0.010383844	2.835147	99.99445847
mGapdh	99.98981517	97.16761	0.007313454
	P0825	M0825	H0825
h SEM	0.001459212	0.091103	0.000421274
m SEM	0.000385351	0.014871	0.004008949

4. Graph ↓

	P0825	M0825	H0825
mGapdh	99.98981517	97.1676133	0.00731345
hGapdh	0.010383844	2.83514667	99.99445847

SEM	P0825	M0825	H0825
mGapdh	0.000385351	0.01487068	0.004008949
hGapdh	0.001459212	0.09110271	0.000421274

$RQ=2^{(-\Delta\Delta Ct)}$

2^6.64386=100

2^9.96578=1000

2^13.2877=10000

5

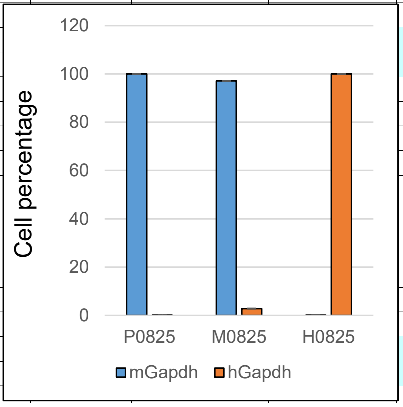

5

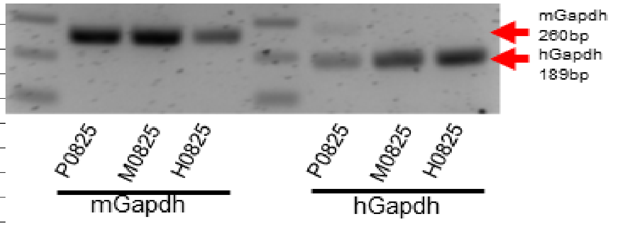

Suppl. Doc 1 (continue)

Block Type	96-Well 0.2-mL Block																	
Chemistry	SYBR_GREEN																	
Date Crea	2021-08-09 17:16:27 PM CDT																	
Experimer	D:\Applied Biosystems\QuantStudio Design & Analysis Software\User Files\experiments\J Jin\2021-08-09_HvsMvsE.eds																	
Experimer	2021-08-09_HvsMvsE																	
Experimer	2021-08-09 23:31:55 PM CDT																	
Experimer	Comparative Cт (ΔΔCт)																	
Instrument	272322662																	
Instrument	272322662																	
Instrument	QuantStudio™ 3 System																	
Passive R	ROX																	
Quantifica	Ct																	
Signal Sm	true																	
Stage whe	Stage3																	
Stage/ Cy	Stage2, Step2																	
Well	Well Po	Sample Na	Target Nan	Reporter	RQ	RQ Min	RQ Max	CT	Ct Mean	Ct SD	Delta Ct M	Delta Ct SD	Delta Ct SE	Delta Delta	Ct Thresh	Baselin	Baseline I	Tm1
1	A1	P0825	mGAPDH	SYBR				19.352	19.384	0.093					0.272	3	12	91.166
2	A2	M0825	mGAPDH	SYBR				18.763	18.776	0.013					0.272	3	12	91.010
3	A3	H0825	mGAPDH	SYBR				32.254	33.730	1.279					0.272	3	25	90.975
13	B1	P0825	mGAPDH	SYBR				19.490	19.384	0.093					0.272	3	13	90.855
14	B2	M0825	mGAPDH	SYBR				18.775	18.776	0.013					0.272	3	13	91.010
15	B3	H0825	mGAPDH	SYBR				34.502	33.730	1.279					0.272	3	27	90.975
25	C1	P0825	mGAPDH	SYBR				19.312	19.384	0.093					0.272	3	14	90.855
26	C2	M0825	mGAPDH	SYBR				18.790	18.776	0.013					0.272	3	13	90.855
27	C3	H0825	mGAPDH	SYBR				34.435	33.730	1.279					0.272	3	26	90.820
37	D1	P0825	hGAPDH	SYBR	1.000	0.658	1.519	33.037	32.648	0.365	13.263	0.376	0.217	0.000	0.268	3	27	84.964
38	D2	M0825	hGAPDH	SYBR	286.570	261.141	314.476	23.957	23.876	0.083	5.100	0.084	0.048	-8.163	0.268	3	18	84.964
39	D3	H0825	hGAPDH	SYBR	##########	########	#########	19.794	19.583	0.184	-14.147	1.292	0.746	-27.410	0.268	3	13	84.935
49	E1	P0825	hGAPDH	SYBR	1.000	0.658	1.519	32.590	32.648	0.365	13.263	0.376	0.217	0.000	0.268	3	26	84.964
50	E2	M0825	hGAPDH	SYBR	286.570	261.141	314.476	23.880	23.876	0.083	5.100	0.084	0.048	-8.163	0.268	3	18	84.809
51	E3	H0825	hGAPDH	SYBR	##########	########	#########	19.501	19.583	0.184	-14.147	1.292	0.746	-27.410	0.268	3	12	84.935
61	F1	P0825	hGAPDH	SYBR	1.000	0.658	1.519	32.315	32.648	0.365	13.263	0.376	0.217	0.000	0.268	3	26	84.499
62	F2	M0825	hGAPDH	SYBR	286.570	261.141	314.476	23.792	23.876	0.083	5.100	0.084	0.048	-8.163	0.268	3	17	84.809
63	F3	H0825	hGAPDH	SYBR	##########	########	#########	19.454	19.583	0.184	-14.147	1.292	0.746	-27.410	0.268	3	13	84.935
Analysis T	Singleplex																	
Endogeno	mGAPDH																	
RQ Min/M	95.0																	
Reference	P0825																	

08/09/2021 SYBR Green qPCR for P0825, M0825 and H0825

Suppl. Doc.2

Cell Line Authentication Service
Mouse STR Profile Report

**Sample Submitted By:**	M.D. Anderson Cancer Center<br>Jiankang Jin
**Email Address:**	jjin@mdanderson.org
**ATCC Sales Order:**	SO1433038
**FTA Barcode:**	MUSA2862
**Cell Line Designation:**	P0825
**Date Sample Received:**	Tuesday, September 06, 2022
**Report Date:**	Tuesday, September 13, 2022

Methodology:

Eighteen Mouse short tandem repeat (STR) loci were analyzed. Two additional markers (Human D8 and D4) to screen for the presence of human or African green monkey species were also utilized. Each sample was processed using the ABI Prism® 3500xl Genetic Analyzer. Data were analyzed using GeneMapper® ID-X v1.2 software (Applied Biosystems). Appropriate positive and negative controls were run and confirmed for each assay set.

Data Interpretation:

Cell lines were authenticated using Short Tandem Repeat (STR) analysis as described in the National Institute of Standards and Technology (NIST) granted U.S. patent (No. 9,556,482). Database matching followed the Tanabe matching algorithm to compare the number of shared alleles between the submitted sample profile and the reference profile, expressed as a percentage.[1]

ATCC performs STR Profiling following ISO 9001:2008 and ISO/IEC 17025:2005 quality standards.
There are no warranties with respect to the services or results supplied, express or implied, including, without limitation, any implied warranty of merchantability or fitness for a particular purpose. ATCC is not is liable for any damages or injuries resulting from receipt and/or improper, inappropriate, negligent or other wrongful use of the test results supplied, and/or from misidentification, misrepresentation, or lack of accuracy of those results. Your exclusive remedy against ATCC and those supplying materials used in the services for any losses or damage of any kind whatsoever, whether in contract, tort, or otherwise, shall be, at the suppliers option, refund of the fee paid for such service or repeat of the service.

The ATCC trademark and trade name, any and all ATCC catalog numbers are trademarks of the American Type Culture Collection. Applied Biosystems, ABI Prism and GeneMapper are registered trademarks of Life Technologies Corporation. All primers used in this assay are described in (US Patent, Pub. No.: US 2017/0101677 A1).

Technical questions?

ATCC Technical Support
(800) 638-6597 / +1 703-365-2700
tech@atcc.org

Ordering questions?

800-638-6597 or 703-365-2700
Fax 703-365-2750
Email: sales@atcc.org

Cell Line Authentication Service
Mouse STR Profile Report

FTA Barcode: MUSA2862

ATCC Sales Order: SO1433038

Test Results for Submitted Sample					ATCC Reference Database Profile			
**Locus**	**Query Profile: MUSA2862**				**Database Profile:**			
**18-3**	18	19						
**4-2**	20.3							
**6-7**	14							
**19-2**	14							
**1-2**	17							
**7-1**	26.2							
**1-1**	11							
**3-2**	13							
**8-1**	13	17						
**2-1**	9	15						
**15-3**	21.3							
**6-4**	18							
**11-2**	15	17						
**17-2**	16							
**12-1**	17							
**5-5**	14	15						
**X-1**	26							
**13-1**	15	18						
Number of shared alleles between query sample and database profile:								NA
Total number of alleles in the query sample profile:								24
Total number of alleles in the database profile:								NA
Percent match between the submitted sample and the database profile:								NA

Explanation of Test Results
Cell lines with ≥80% match are considered to be related; i.e., derived from a common ancestry. Cell lines with a percent match between a 55 - 80% require further investigation for authentication of relatedness.

☐ The submitted sample profile is an exact match for the following ATCC cell line(s) in the ATCC mouse STR database:

☑ The submitted sample profile is mouse, however a matching reference profile has not previously been established in the ATCC mouse STR database.

☐ The submitted profile is similar to the following ATCC cell line(s):

☐ An STR profile could not be generated from the submitted sample.

Human and/or African Green Monkey Species Detection

☐ Human and/or African green monkey has been detected in the submitted sample profile (see attached electropherogram at Human D8 & D4 loci).

Additional Comments:

N/A

Cell Line Authentication Service
Mouse STR Profile Report

Addendum: Comparative Output from the ATCC STR Profile Database

FTA Barcode: MUSA2862

ATCC Sales Order: SO1433038

% Match	100
ATCC® Cat. No.	MUSA2862
Designation	P0825
18-3	18,19
4-2	20.3
6-7	14
19-2	14
1-2	17
7-1	26.2
1-1	11
3-2	13
8-1	13,17
2-1	9,15
15-3	21.3
6-4	18
11-2	15,17
17-2	16
12-1	17
5-5	14,15
X-1	26
13-1	15,18

Definitions of terms used in this report:

Artifact:
A non-allelic product of the amplification process, an anomaly of the detection process, or a by-product of primer synthesis.

Pull-up:
A term used to describe when signal from one dye color channel produces artificial peaks in another, usually adjacent, color.

Spike:
An extraneous peak resulting from dust, dried polymer, an air bubble, or an electrical surge.

Dye blob:
Free dye not coupled to primer that can be injected into the capillary.

e-Signature, Technician:	jbrowne 9/12/2022
e-Signature, Reviewer:	Bchase 9/13/2022

Reference:
1. Tanabe H, et al. Cell line individualization by STR multiplex system in the cell bank found cross-contamination between ECV304 and EJ-1/T24. Tiss Cult Res Commun 18:329-38, 1999.

ATCC® | Credible leads to Incredible™

27 September 2022

Re: Mouse Cell STR Profiling Service

Sales order SO13433038
MUSA2862

To whom it may concern;

ATCC currently has approximately 400 mouse cell lines that have been profiled and are in our database. The sample MUSA2862 shows no signs of contamination. The profile generated from sample MUSA2862 appears to have come from a single cell line, which is unique in that it does not match any profile in our database.

ATCC is committed to provide quality products and services to the life science community. The commitment includes the following certifications and accreditations: ISO 9001 Certified, ISO/IEC 17025 Accredited, ISO 13485 Certified, and ISO 17034 Accredited. The processes surrounding the provision of products and services are monitored to ensure that product quality objectives and customer needs are met. This very high quality level is the best available practice in this industry.

For over 90 years, ATCC has been a non-profit supplier of biomaterials to the scientific community for government, industry, education, healthcare, and research laboratories, with a well-known reputation for the highest quality products. Many of our cultures and products are used as standards by which other products and procedures are measured.

If you have any questions or concerns pertaining to this letter, please do not hesitate to contact our Technical Service Department at via email to tech@atcc.org .

American Type Culture Collection (ATCC®)
P.O. Box 1549
Manassas, VA 20110 USA
www.atcc.org

(800) 638-6597 or 703-365-2700
Fax: 703-365-2750
Doc ID: 8409
Effective Date: 03/28/2019
Revision: 7

Sample Name	CGQ
MUSA2862_P0825	

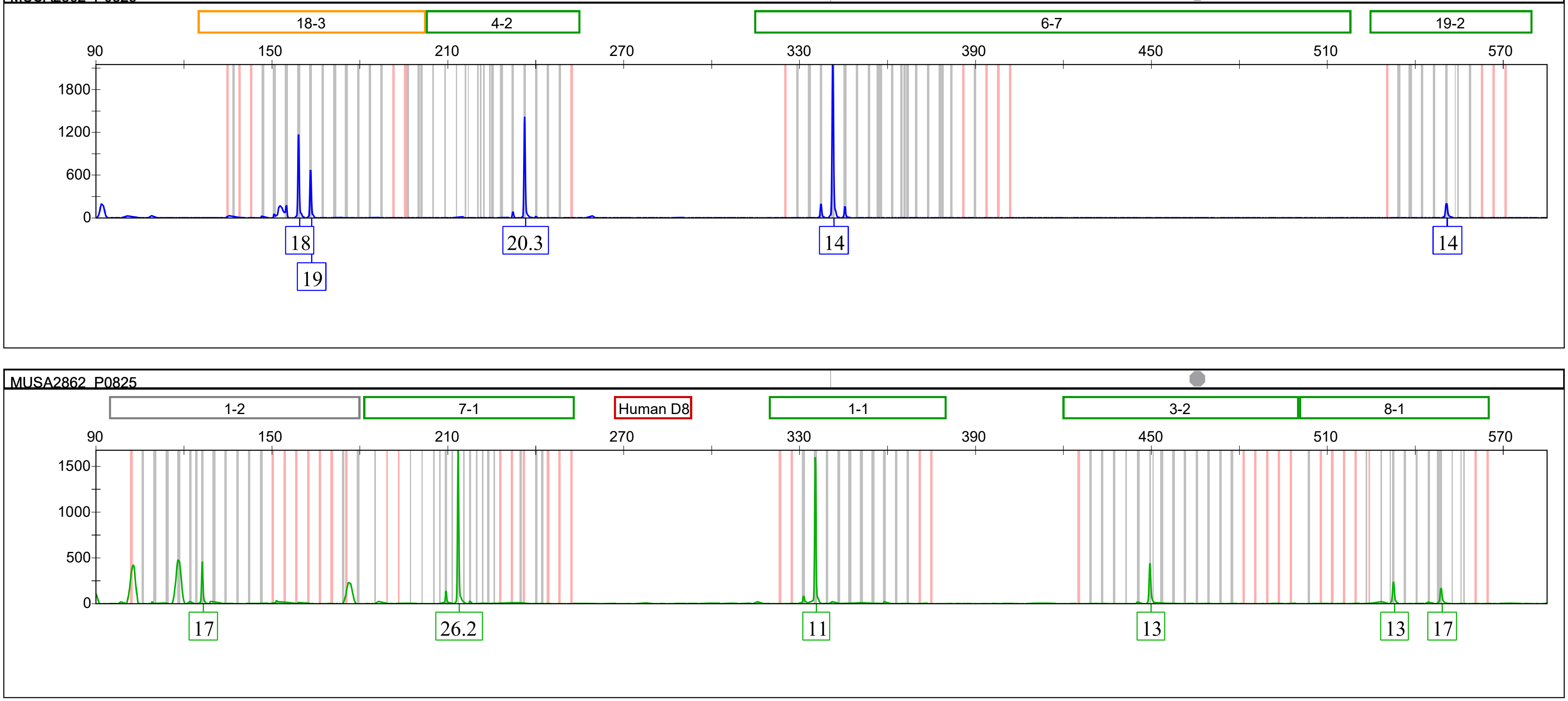

Sample Name	CGQ
MUSA2862_P0825	

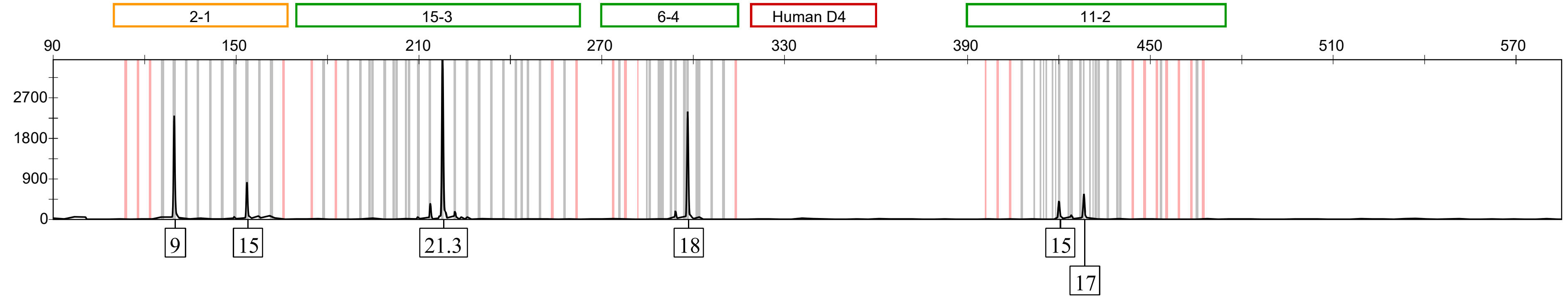

MUSA2862_P0825	

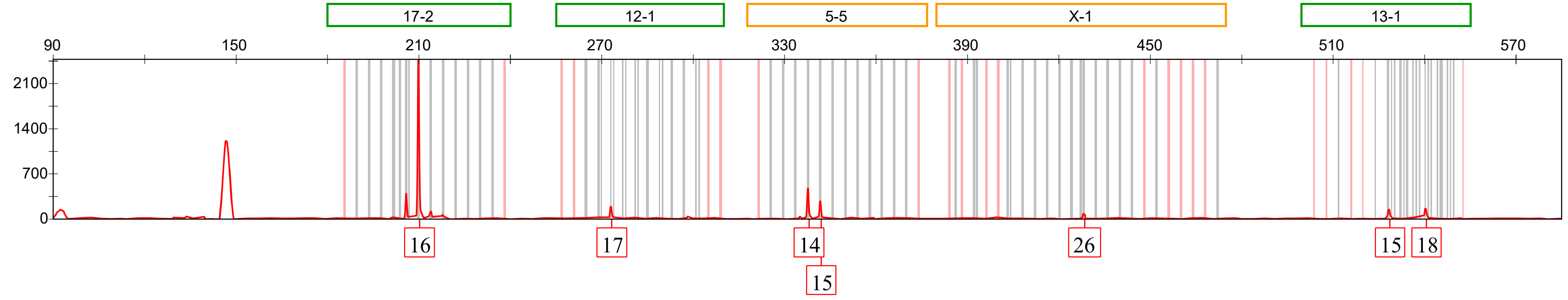

Supplement: Supplementary file 1 [file DataSheet_1.pdf]
